# An integrated approach to improve plant protection against olive anthracnose caused by the *Colletotrichum acutatum* species complex

**DOI:** 10.1371/journal.pone.0233916

**Published:** 2020-05-29

**Authors:** Stefanos Kolainis, Anastasia Koletti, Maira Lykogianni, Dimitra Karamanou, Danai Gkizi, Sotirios E. Tjamos, Antonios Paraskeuopoulos, Konstantinos A. Aliferis

**Affiliations:** 1 Laboratory of Pesticide Science, Agricultural University of Athens, Athens, Greece; 2 Laboratory of Biological Control of Pesticides, Benaki Phytopathological Institute, Kifissia, Greece; 3 Laboratory of Plant Pathology, Agricultural University of Athens, Athens, Greece; 4 Directorate of Rural Economy and Veterinary of Trifilia, Prefecture of Peloponnese, Kyparissia, Greece; 5 Department of Plant Science, Ste-Anne-de-Bellevue, QC, Canada; Fujian Agriculture and Forestry University, CHINA

## Abstract

The olive tree (*Olea europaea* L.) is the most important oil-producing crop of the Mediterranean basin. However, although plant protection measures are regularly applied, disease outbreaks represent an obstacle towards the further development of the sector. Therefore, there is an urge for the improvement of plant protection strategies based on information acquired by the implementation of advanced methodologies. Recently, heavy fungal infections of olive fruits have been recorded in major olive-producing areas of Greece causing devastating yield losses. Thus, initially, we have undertaken the task to identify their causal agent(s) and assess their pathogenicity and sensitivity to fungicides. The disease was identified as the olive anthracnose, and although *Colletotrichum gloeosporioides* and *Colletotrichum acutatum* species complexes are the two major causes, the obtained results confirmed that in Southern Greece the latter is the main causal agent. The obtained isolates were grouped into eight morphotypes based on their phenotypes, which differ in their sensitivities to fungicides and pathogenicity. The triazoles difenoconazole and tebuconazole were more toxic than the strobilurins being tested. Furthermore, a GC/EI/MS metabolomics model was developed for the robust chemotaxonomy of the isolates and the dissection of differences between their endo-metabolomes, which could explain the obtained phenotypes. The corresponding metabolites-biomarkers for the discrimination between morphotypes were discovered, with the most important ones being the amino acids L-tyrosine, L-phenylalanine, and L-proline, the disaccharide *α*,*α*-trehalose, and the phytotoxic pathogenesis-related metabolite hydroxyphenylacetate. These metabolites play important roles in fungal metabolism, pathogenesis, and stress responses. The study adds critical information that could be further exploited to combat olive anthracnose through its monitoring and the design of improved, customized plant protection strategies. Also, results suggest the necessity for the comprehensive mapping of the *C*. *acutatum* species complex morphotypes in order to avoid issues such as the development of fungicide-resistant genotypes.

## 1. Introduction

**Fig 1:**
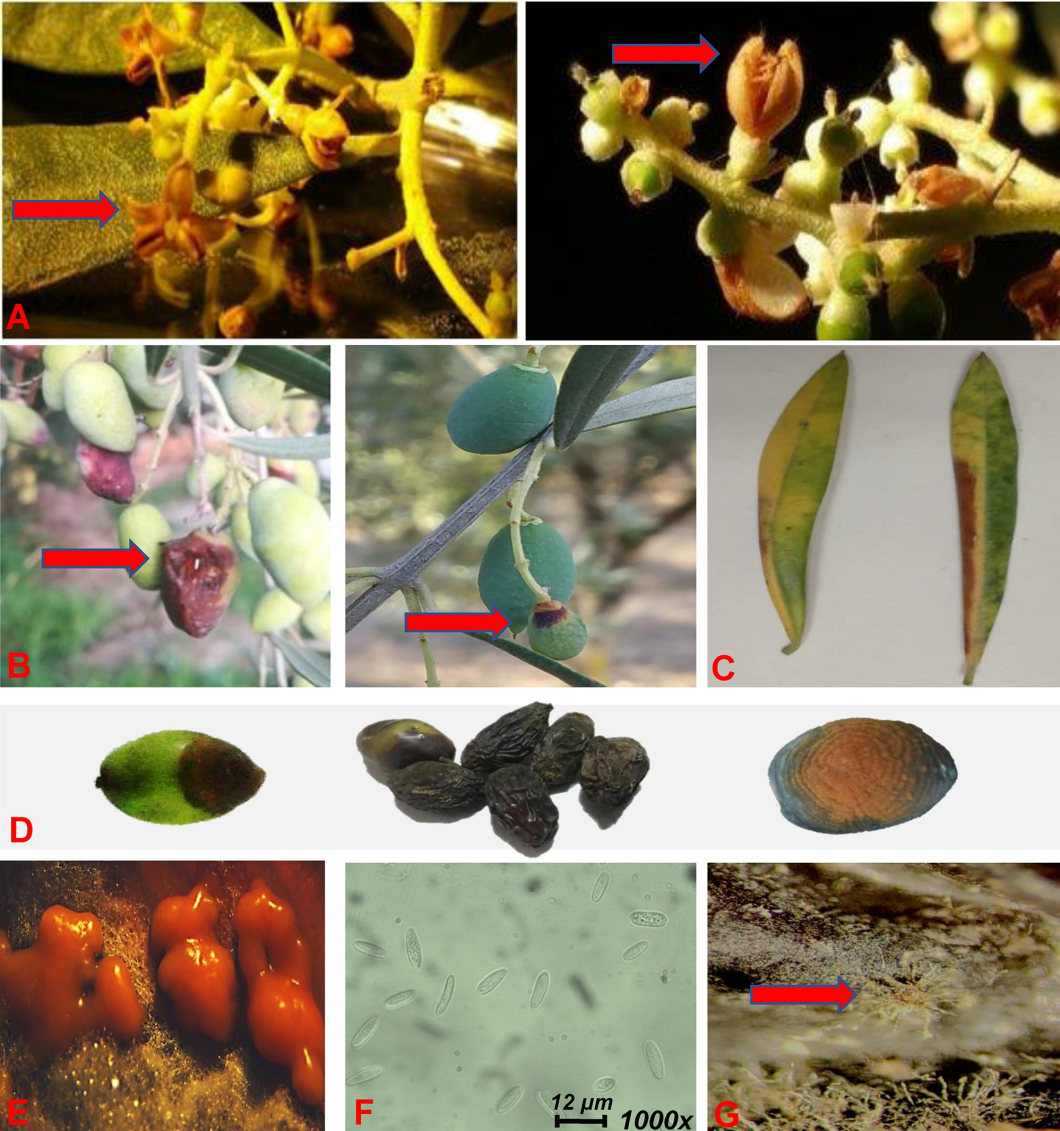
Symptoms of olive anthracnose on olive fruits, flowers and leaves, and the causal pathogen. Typical symptoms of olive anthracnose caused by *Colletotrichum acutatum* on olive flowers (artificially infected, red arrow) (**A**), fruits (red arrow) (**B,D**), and leafs (**C**). Gelatinous masses (conidiomata) (**E**) of spores (**F**) appear on the top of cultures grown on PDA, and acervuli (red arrow) developed on infected fruits (**G**).

The olive tree (*Olea europaea* L.) has been cultivated for millennia, being the most important oil-producing crop of the Mediterranean basin [1] and vital source of revenue for the local societies. In addition to the superior organoleptic properties of olive oil [2], the olive tree cultivation is gaining popularity due to the increasing awareness of the public on the health benefits associated to its oil consumption [3–7]. Nonetheless, periodic outbreaks of emerging diseases, often lead to severe yield losses, with the most devastating ones caused by fungi of the genera *Colletotrichum* spp. (olive anthracnose) (**Fig 1**), *Spilocaea oleagina* (peacock spot), and *Cladosporium* sp. (cladosporium rot) [7].

During the past 3 years in Greece, especially in the Peloponnese prefecture, one of the major olive-producing areas, there has been an outbreak of a disease that causes symptoms in olive fruits consistent with biotic infections, resembling those caused by *Colletotrichum* spp. [8] (**Fig 1**). Based on our estimations, the disease results in an average of 300 million € worth of product losses yearly, thus, it is considered as an emerging threat for the sector. Although the cause of such outbreaks is yet to be investigated, interestingly, a strong correlation has been observed between the environmental conditions in the affected areas (e.g. increased relative humidity and rain levels, and abnormal temperature fluctuations) and the disease severity. The first report on the disease in Greece is dated in 1920 for the Ionian island of Corfu, where a serious outbreak was recorded [9]. Currently, the disease is being detected in almost all major olive producing regions of the country, and recently has been reported in parts of Western Greece [8].

The olive anthracnose (*Colletotrichum* spp., teleomorph: *Glomerella*, Glomerellaceae), also known as pastel, is considered as the most devastating fungal disease of olive trees [10, 11], especially in areas with high levels of relative humidity [10]. In many cases, heavy infections can even eliminate the production [12]. The disease was initially reported in Portugal in 1899 by J.V. d'Almeida [13] and later on in Spain (known as “fruit soap”), and Italy, known as “leprosy” [14–16]. Currently, there are reports on its occurrence in almost all major olive oil-producing areas of the Mediterranean basin [17–20]. Nonetheless, the genus *Colletotrichum* spp. has a worldwide distribution [20–26] infecting a large variety of hosts [27], ranking as the 8^th^ most scientifically/economically important group of plant pathogenic fungi [28].

*Colletotrichum* spp., is a complex species, with *Colletotrichum gloeosporioides sensu lato (s*.*l*.*)* (e.g. *C*. *aenigma*, *C*. *gloeosporioides s*.*s*., *C*. *kahawae*, *C*. *siamense*) and *Colletotrichum acutatum (s*.*l*.*)* [e.g. *C*. *godetiae*, *C*. *acutatum sensu stricto (s*.*s*.*)*, *C*. *nymphaeae*, *C*. *rhombiforme*] being the main species responsible for olive anthracnose [12, 29, 30]. However, in Portugal, the latter has been discovered as the predominant species, with >97% occurrence in infected fruits, followed by the former (<3%) [19]. Similarly, the *C*. *acutatum* species complex was discovered as the predominant in infected fruits in Italy [30].

The pathogen mainly infects olive fruits (**Fig 1B, 1D and 1G**) causing severe yield losses, but flower infections can also be observed (**Fig 1A**). In varieties with elongated fruit shape, infections usually begin at their tips, while in those with large, spherical fruit shape, they appear as small necrotic spots, which evolve rapidly (**Fig 1D**). The infected tissues shortly are covered by fungal spore masses (**Fig 1E and 1F**) and new infections mainly occur late in fall [30]. In advanced stages of infection, the fruits fall or remaining on the trees and get mummified (**Fig 1D**). The latter are considered as the main sources of inocula for the infections that take place early in the spring (**Fig 1A**) [31]. Mature or near-maturation fruits are the most vulnerable, but under favorable environmental conditions, infections can also occur at early developmental stages (**Fig 1B**). The infected flowers initially wilt and then dry (**Fig 1A**). However, it is possible to remain asymptomatically [32], and the pathogen may stay dormant [14]. Less frequent are infections of fruit stems, young branches, and leaves (**Fig 1C**). The optimum growth temperature ranges between 20 and 27°C.

The effect of olive anthracnose on olive trees and the olive oil production is devastating, although various fungicides are available (**S1 Table**) and plant protection measures are regularly applied. The plant protection against the disease is based on integrated pest management (IPM) strategies, including the use of chemical plant protection products (PPPs) with copper-based fungicides to be the most commonly applied ones [32], resistant cultivars [11], and early harvesting [10]. However, the ineffectiveness to control the disease dictates the need that further research is required in order to acquire relative knowledge that could be exploited in the design of optimized, advanced plant protection strategies. Recently, the potential of yeasts as biological control agents against *C*. *gloeosporioides* [33] and pomegranate peel extract against *C*. *acutatum* has been highlighted [34].

Within this context, initially, we have undertaken the task to identify the causal agent of olive fruit infections in Southern Greece, and assess the pathogenicity of the fungal pathogen(s) and their sensitivity to selected active ingredients (a.i.) of fungicides that are registered for the plant protection of olive trees. Additionally, based on the capabilities of GC/EI/MS metabolomics in fungal chemotaxonomy [35, 36], a model was additionally developed for the robust chemotaxonomy of *C*. *acutatum* species complex isolates, and the dissection of the differences in their endo-metabolomes, which could probably explain the observed niches. To the best of our knowledge no reports exist on the resistance of *Colletotrichum* spp. morphotypes to fungicides, and no related metabolomics studies have been performed.

## 2. Materials and methods

### 2.1. Chemicals and reagents

The a.i. of the fungicides being assessed were of technical grade purity; pyraclostrobin (99.9%, w/w) and kresoxim-methyl (92%, w/w) were kindly provided by BASF Crop Protection (Ludwigshafen, Germany), difenoconazole (99.3%, w/w) by Syngenta International AG (Basel, Switzerland), and trifloxystrobin (99.9%, w/w) and tebuconazole, by Bayer CropScience AG (Leverkusen, Germany). The antibiotic enrofloxacin was also provided by Bayer CropScience AG. The solvents ethyl acetate and methanol that were used in the extraction of the fungal endo-metabolome, were of GC/MS grade (99.9% purity, Carlo Erba Reagents, val de Reuil, France). Reagents for GC/EI/MS metabolomics analysis such as, pyridine (99.8%, v/v), methoxylamine hydrochloride (98%, w/w), N-methyl-N-(trimethyl-silyl) trifluoroacetamide (MSTFA), and analytical standards of selected metabolites, were purchased from Sigma-Aldrich Ltd (Steinheim, Germany). Ribitol (Sigma-Aldrich Ltd), was used as the internal standard.

### 2.2. Plant material

Olive fruits of the variety “Koroneiki” with visible symptoms of infections were sampled from olive orchards of the Peloponnese prefecture (**S1 Fig**). More specifically, samples were collected from olive orchards of the greater Kyparissia (Messinia, 37.2512°N and 21.6694°E) and Gytheio (Laconia, 36.7610°N and 22.5640°E) areas, which are among the major olive-producing regions of the prefecture. The sampling took place during the period of fall-winter of 2016-’17 and 2017-’18. Samples were collected into plastic bags, labeled properly, and sent overnight to the Laboratory of Pesticide Science (Agricultural University of Athens, AUA). Forty samples of approximately 60 gr each, were collected per season (80 in total).

Their processing was performed within 24 h from their receipt. In the bioassays for the assessment of the pathogenicity of the obtained fungal isolates, fresh, healthy, and mature surface-sterilized fruits and flowers of the cultivars “Koroneiki” and “Kalamon”, were used.

### 2.3. Isolation of fungi from infected olive fruits and growth conditions

Infected olive fruits showing variable symptoms of infection (**Fig 1B and 1D**), were grouped and coded. Their surface sterilization followed; initially, immersion in 100% ethanol (1 min), then, in 5% sodium hypochlorite solution (4 min), followed by immersion in 100% ethanol (30 sec), and finally, rinsing with sterile deionized water (four times). Prior to their sterilization, the fruits were placed for 15 sec on PDA (Becton, Dickinson and Company, Le Pont de Claix, France), in 9 cm-in diameter Petri plates, in order to record their imprints. Such practice assists the separation between epi- and endophytic microbes and the assessment of the efficacy of the sterilization protocol and practices [37]. Then, an incision was made at the center of the necrotic lesion(s) of each sample, followed by observations under a stereomicroscope, in order to detect possible infections by pests such as those caused by the olive fly *Bactroceraoleae* (Rossi) (Diptera: Tephritidae) [38]. In just a handful of samples pest infections were observed, which were then discarded, since the observed symptoms could be the result of secondary fungal infections.

Minute rectangular portions of the fruits, including epicarp and mesocarp tissues, were then removed and placed on PDA containing the antibiotic enrofloxacin (2 μgmL^-1^), in Petri plates, and incubated at 22±1°C in the dark. The samples were taken from the edges of the necrotic lesions and contained parts of visually healthy tissue. Upon the observation of hyphal growth, small portions were aseptically taken, transferred into new media (PDA), and incubated. A unique code was given to each of the obtained pure cultures, which were registered at the Pesticide Metabolomics Group (PMG) Database (AUA). Sub-culturing was performed bi-weekly using 4-mm in diameter mycelial discs taken from the edges of the cultures. Additionally, for validation purposes a *C*. *gloeosporioides* isolate, which was kindly provided by Professor D. Tsitsigiannis (Laboratory of Plant Pathology, Agricultural University of Athens), was subjected to PCR analysis.

### 2.4. Phenotyping, tentative, and absolute identification of fungal isolates

#### 2.4.1. Phenotyping and tentative identification

The phenotypes of the obtained pure cultures of the isolates were documented, and hyphal and spore samples, when available, were microscopically examined. The tentative identification of the isolates was performed based on the phenotypic characteristics of the conidia and that of the cultures (shape, color and density of hyphae) using taxonomic keys [39].

#### 2.4.2. Identification of the isolated strains using molecular markers

Based on the results of the tentative identification of isolates, it was concluded that their majority belong to *Colletotrichum* spp. Furthermore, since studies have suggested the prevalence of *C*. *acutatum* species complex in many Mediterranean countries [19, 30, 40, 41], initially, our effort was focused on testing whether the obtained isolates belong to *C*. *acutatum*. For this purpose, specific primers that bind at the conserved regions of the *β*-tubulin gene and internal transcribed spacer (ITS) region of ribosomal DNA of *C*. *acutatum* [19] (Eurofins Genomics, Luxembourg, Luxembourg), were used (Table 1). Additionally, for validation purposes, a second PCR analysis was performed using the corresponding primers for *C*. *gloeosporioides* (Table 1). In the analysis, the 17 tentatively identified as *C*. *acutatum* isolates and the *C*. *gloeosporioides* isolate, were included.

**Table 1:** Specific primers that were used in the identification of *Colletotrichum acutatum* and *Colletotrichum gloeosporioides* isolates.

*Amplification target*	*Primers*	*Product*
***β*-tubulin**	*Colletotrichum acutatum*	
Forward	TBCA (5’-CGGAGGCCTGGTTGGGTGAG-3’)	
		300bp
Reverse	TB5 (5’-GGTAACCAGATTGGTGCTGCCTT-3’)	


	*Colletotrichum gloeosporioides*	300bp
Forward	TBCG (5’-CGGAAGCCTGGGTAGGAGCG 3’)	
Reverse	TB5 (5’-GGTAACCAGATTGGTGCTGCCTT-3’)	
**ITS region**	*Colletotrichum acutatum*	
Forward	CaInt 2 (5’-GGGGAAGCCTCTCGCGG-3’)	
		500 bp
Reverse	ITS4 (3’-TCCTCCGCTTATTGATATGC-5’)	

	*Colletotrichum gloeosporioides*	
		500 bp
Forward	CgInt (GGCCTCCCGCCTCCGGGCGG)	
Reverse	ITS4 (3’-TCCTCCGCTTATTGATATGC-5’)	


Fresh cultures of the 17 tentatively identified as *Colletotrichum* spp. groups of isolates were grown on PDA, at 22±1°C, in the dark. Samples were taken 7 days following their inoculation for DNA extraction [42]. Briefly, the concentration of the retained DNA and its purity were measured using a Nanodrop spectrophotometer ND-2000C (Thermo Scientific, Waltham, MA, U.S.A.). The samples were then diluted in double-distilled, sterilized water to a final concentration of 50 ng mL^-1^. The targeted genes were amplified by polymerase chain reaction (PCR, Applied Biosystems Veriti^TM^ 96-Well Thermal Cycler, Thermo Scientific), using the selected primers [19]. Each reaction was carried out in a final volume of 10 μL using the DreamTaq Green PCR Master Mix (2X) (K1081, Thermo Scientific). The thermal cycles started with denaturation at 95°C for 4 min, followed by 35 cycles of 95°C for 30 sec, 62°C for 30 sec and 72°C for 30 sec, and ended with 8°C. Visualization of the amplicon was performed by electrophoresis on 1% agarose gels using LumiBIS 1.4 (DNR Bio-Imaging Systems Ltd,NeveYamin, Israel).The molecular marker GeneRuler 1kbDNAladder (Thermo Fisher Scientific) was used.

### 2.5. Assessment of the sensitivity of *Colletotrichum acutatum* species complex isolates to selected fungicides that are registered in the plant protection of olive trees

In preliminary experiments, the sensitivity of isolates of various morphotypes (M), that were molecularly identified as *C*. *acutatum* to selected a.i., had been assessed (data not shown). The a.i. difenoconazole and tebuconazole (triazoles), and kresoxim-methyl, pyraclostrobin, and trifloxystrobin (strobilurins), which are registered and widely used in the plant protection of olive trees (**S1 Table**), were selected. Based on the results, isolates of the M1-M8, were chosen on the basis of their sensitivity to the a.i. Working stock solutions of the fungicides were prepared in methanol (GC/MS grade, 99.9% purity).

The fungal isolates were grown on PDA amended with the selected triazoles at the concentrations of 0.1, 1, 5, and 15 μg mL^-1^, whereas for strobilurins the concentration of 30 μg mL^-1^ was additionally assessed. Cultures grown on PDA in which quantities of methanol equal to those added with the highest concentration of a.i., served as controls. Four biological replications were performed per treatment, and two mycelial plugs were added symmetrically in each plate. The assessment of the bioactivity of the a.i. was based on their effect on the radial growth of the cultures seven days following treatments. Measurements were taken using the software ImageJ [43] and statistical analysis was performed using the software JMP v.13 (SAS Institute Inc., Cary, NC) applying the Student’s *t*-Test (*α* = 0.05).

### 2.6. Assessment of the pathogenicity of selected *Colletotrichum acutatum* complex species strains to olive fruits and flowers

For the assessment of the pathogenicity of selected *C*. *acutatum* complex species strains, fresh and healthy olive fruits and flowers of the varieties “Koroneiki” and “Kalamon, were used. These two cultivars account for the vast majority of cultivated olive trees in Greece. In the bioassays, the isolates PLS_90 (M1) and PLS_88 (M2), were selected, the former being sensitive to a.i. employed in the plant protection of olive trees, and the latter resistant.

A previously described protocol was adapted [14]; the fruits were initially surface-sterilized, and the flowers were carefully examined in order to detect possible infections. Freshly prepared aquatic spore suspensions that had been harvested from 7-day old cultures grown in PDA, served as the inocula. The density of the suspensions was adjusted to 10^6^spores mL^-1^, using a hemocytometer (Improved Neubauer, Hirschmann Laborgeräte GmbH & Co. KG, Eberstadt, Germany).

In order to test the hypothesis that the pathogens can infect both healthy and priory infected by pests fruits, a droplet of the spore suspensions (15 μL) was placed on intact epicarps, or fruits whose epicarp had been mechanically injured (punctured). One droplet was added per fruit, whereas sterilized water served as the control. The fruits were attached in plastic 9 cm-in diameter Petri plates, and incubated in a box, in the dark, at 22±1°C, and observations were taken in 24 h intervals. At the bottom of the box, a moistened sterilized filter paper was placed in order to maintain the relative humidity at high levels. Similarly, droplets were added on freshly cut and healthy flowers, which were treated as described above for the olive fruits.

### 2.7. Gas chromatography/electron impact/mass spectrometry (GC/EI/MS) for metabolite profiling of the *Colletotrichum acutatum* species complex endo-metabolome and chemotaxonomy

#### 2.7.1. Experimental design and sample preparation for metabolomics analyses

The *C*. *acutatum s*pecies complex isolates PLS_82 (M5), PLS_88 (M2), PLS_90 (M1), PLS_91 (M4), PLS_111 (M1), PLS_85 (M8), PLS_92 (M3), and PLS_93 (M4), were grown in Petri plates (9 cm-in diameter) containing 15 mL of PDA. Their selection was made on the basis of their M and results on their sensitivity to the applied a.i. Inocula for the initiation of the cultures were taken from 7-day old cultures. In order to be able to analyze the fungal endo-metabolome, prior to media inoculation, a sterile cellophane membrane (500PUT, Futamura USA Inc., Atlanta, GA, USA) was placed on the surface of the solid media. The incubation of cultures was performed at 22±1°C, in the dark.

Seven days following inoculations of the media, hyphae were scrapped off and collected into plastic Eppendorf tubes (2 mL). The quenching of their metabolism was immediately performed by immersing the tubes into liquid N_2_. The pulverization of hyphae to a fine powder in a mortar with a pestle under liquid N_2_, followed. For metabolomics analyses, 40 mg of fresh weight (FW) of the pulverized hyphae were placed into glass autosampler vials (2 mL, Macherey-Nagel, Duren, Germany), while the rest was stored at -80°C until further use. In total, 15 biological replications were performed per isolate, which were finally pooled in groups of three, to finally obtain five pooled samples. The extraction of the fungal endo-metabolome and the derivatization of metabolites were performed as previously described, with minor modifications [44]. For details please consult the **S1 File** section.

#### 2.7.2. Settings of gas chromatography/electron impact/mass spectrometry (GC/EI/MS) metabolomics analyses

In the analysis, an Agilent 6890 MS analytical platform (Agilent Technologies Inc., Santa Clara, CA, USA), was employed. The platform was equipped with the 7683 automatic inert mass selective detector (MSD) and an HP-5MS ultra inert (UI) capillary column (30 m, i.d. 0.25 mm, film thickness 0.25 μm, Agilent Technologies Inc.), was used. All experimental events were controlled by the software MSD ChemStation v.E.02.01.11.77 (Agilent). Previously described settings were used in the analyses [44, 45]. For a detailed description please consult the S1 File section.

#### 2.7.3. Deconvolution of the *Colletotrichum acutatum* endo-metabolome

The acquired GC/EI/MS chromatograms were deconvoluted using the AMDIS software v.2.66 (NIST; Gaithersburg, MD, USA). The putative identification of metabolite features was based on matching their mass spectra to entries of the National Institute of Standards and Technology library, NIST 08 (NIST; Gaithersburg, MD, USA). Peaks present also in the blank samples were removed from further analyses. The absolute identification of selected metabolites was performed by matching the mass spectra and retention times to analytical standards that had been analyzed in the same analyzer following the same method, as proposed by the Metabolomics Standards Initiative (MSI) [46].

#### 2.7.4. Chemotaxonomy and discovery of the metabolite differences between the endo-metabolomes of the analyzed *Colletotrichum acutatum* species complex morphotypes

The discovery of trends and the corresponding biomarkers of *C*. *acutatum* species complex isolates, was based on previously described protocols [44, 45] with a few modifications. The processing of the acquired raw GC/EI/MS chromatograms was performed using the software MS-DIAL v.3.70 [47]. The resulting matrix was subjected to multivariate analyses using bioinformatics software SIMCA-P + v.13.0.3 (Umetrics, Sartorius Stedim Biotech, Umeå, Sweden) for the discovery of trends and the corresponding biomarkers. Orthogonal partial least squares-discriminant analysis (OPLS-DA) and OPLS-hierarchical cluster analysis (OPLS-HCA) was performed for an initial overview of the data set. The discovery of the differences between the endo-metabolomes of the analyzed isolates was based on the contribution plots.

## 3. Results and discussion

### 3.1. Various fungal isolates were obtained from the infected olive fruits exhibiting morphological features consistent with those of *Colletotrichum* spp.

From the infected olive fruit samples originating from various locations of Southern Greece (**S1 Fig**), thirty seven fungal isolates were obtained, which were further grouped based on phenotypic, stereomicroscopic, and microscopic observations into 28groups. Among the isolated strains, seventeen were tentatively identified as *Colletotrichum* spp., based on their phenotypes, sporulation, and spores’ morphology (**S2 Table**), using taxonomic keys, and they were grouped in eight morphotypes. These isolates produce numerous spores on their surface, appeared as a pink, yellow, or orange gelatinous masses (**Fig 1E**). The mature spores had fusiform, elliptical, or cylindrical shape and variable dimensions; average length 12.26μm, average width 4.17μm (**Fig 3 and S2 Table**), features, which are all consistent with those of *Colletotrichum* spp. The slow growth rate of their cultures, was another common feature of the isolates. The complexity of the *Colletotrichum* spp. [27, 48], makes the further identification challenging, requiring the use of molecular markers. The remaining isolates (**S3 Table**) had features not consistent with *Colletotrichum* spp., probably they were secondary infections of others pathogens related to olive trees such as *S*. *oleagina*, *Cladosporium* sp., *Botryosphaeria dothidea*, since most of them had been isolated from severely infected (mummified) fruits or even endophytic or saprophytic fungi. Therefore, they were excluded from further analysis. Furthermore, for species such as *S*. *oleagina* the applied protocol is not effective for the isolation of the pathogen (e.g. long time is required, and its isolation from plant tissues is challenging).

### 3.2. The *Colletotrichum acutatum* species complex is the main cause of the severe yield losses of olive oil and fruits that have been observed in major olive-producing areas of Southern Greece

**Fig 2:**
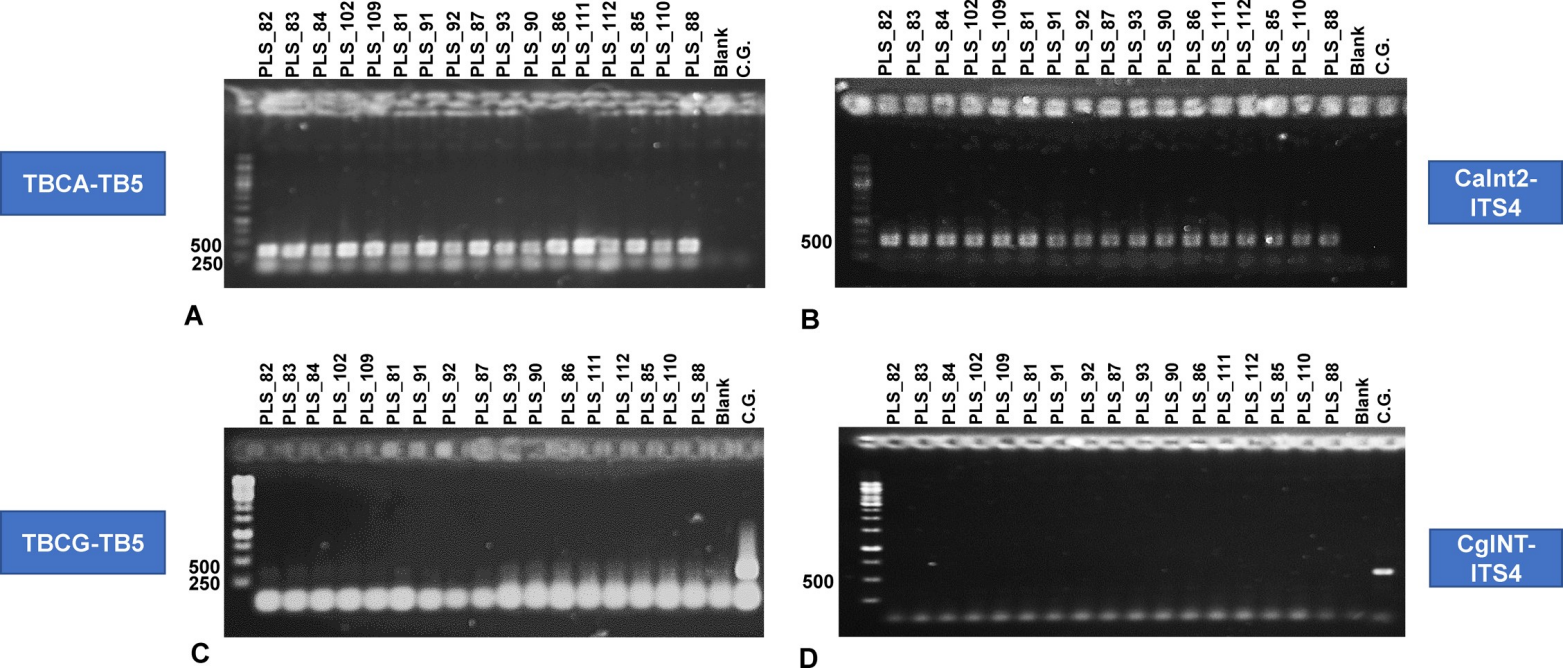
Agarose gel electrophoresis of PCR products following amplification with species-specific primers for *Colletotrichum acutatum* species complex and *Colletotrichum gloeosporioides* isolates. Products obtained using primers for the *C*. *acutatum β*-tubulin gene (forward primer: TBCA, reverse primer: TB5) (A) and the ITS region (forward: CaInt2, reverse ITS4) (B), and the *C*. *gloeosporioides β*-tubulin gene (forward primer: TBCG, reverse primer: TB5) (C) and the ITS region (forward: CgInt, reverse ITS4) (D), on 1% agarose gel.

The first major finding of the research is that all isolates that had been obtained from the infected fruits from Southern Greece (Peloponnese), were identified as *C*. *acutatum*. Such observation confirms that fungi of the complex is the predominant cause of olive anthracnose, which has led to the recent severe quantitative and qualitative yield losses of olive oil and fruits. The isolates were initially tested for their affinity to *C*. *acutatum* using species-specific primers that amplify regions in the conserved tubulin and ITS genes [19]. The reaction products were electrophoresed, and bands corresponding to the expected sizes of the primer products, 500 bp for the ITS region, and between 300–400 bp for the tubulin gene, were observed (**Fig 2**). Due to the simultaneous amplification of the two conserved genes, it was confirmed that the strains belong to the *C*. *acutatum* species complex. The inclusion of the *C*. *gloeosporioides* isolate and the utilization of the corresponding species-specific primers further confirmed that all the isolates being studied were indeed belonged to *C*. *acutatum* species complex and the validity of the applied protocol in identifying and discriminating between the two major olive anthracnose species.

To the best of our knowledge, this is the first report on the *C*. *acutatum* species complex as the dominant species causing the disease olive anthracnose in Southern Greece. Such observation is in accordance with previous research, which have reported that the *C*. *acutatum* species complex is the dominant *Colletotrichum* species in Italy [30], Spain [40], Tunisia [41], and Portugal [19], responsible for olive anthracnose. In both Tunisia and Portugal, its occurrence in infected fruits was more than >95–97%, followed by *C*. *gloeosporioides s*.*l*. with 3–5%. Therefore, although both *C*. *gloeosporioides* and *C*. *acutatum* are known to cause olive anthracnose [12, 27–29], the current evidence suggest that, in the Mediterranean countries, the latter is the major causal agent of the disease.

### 3.3. The obtained *Colletotrichum acutatum* species complex isolates are grouped in eight major morphotypes (M)

**Fig 3:**
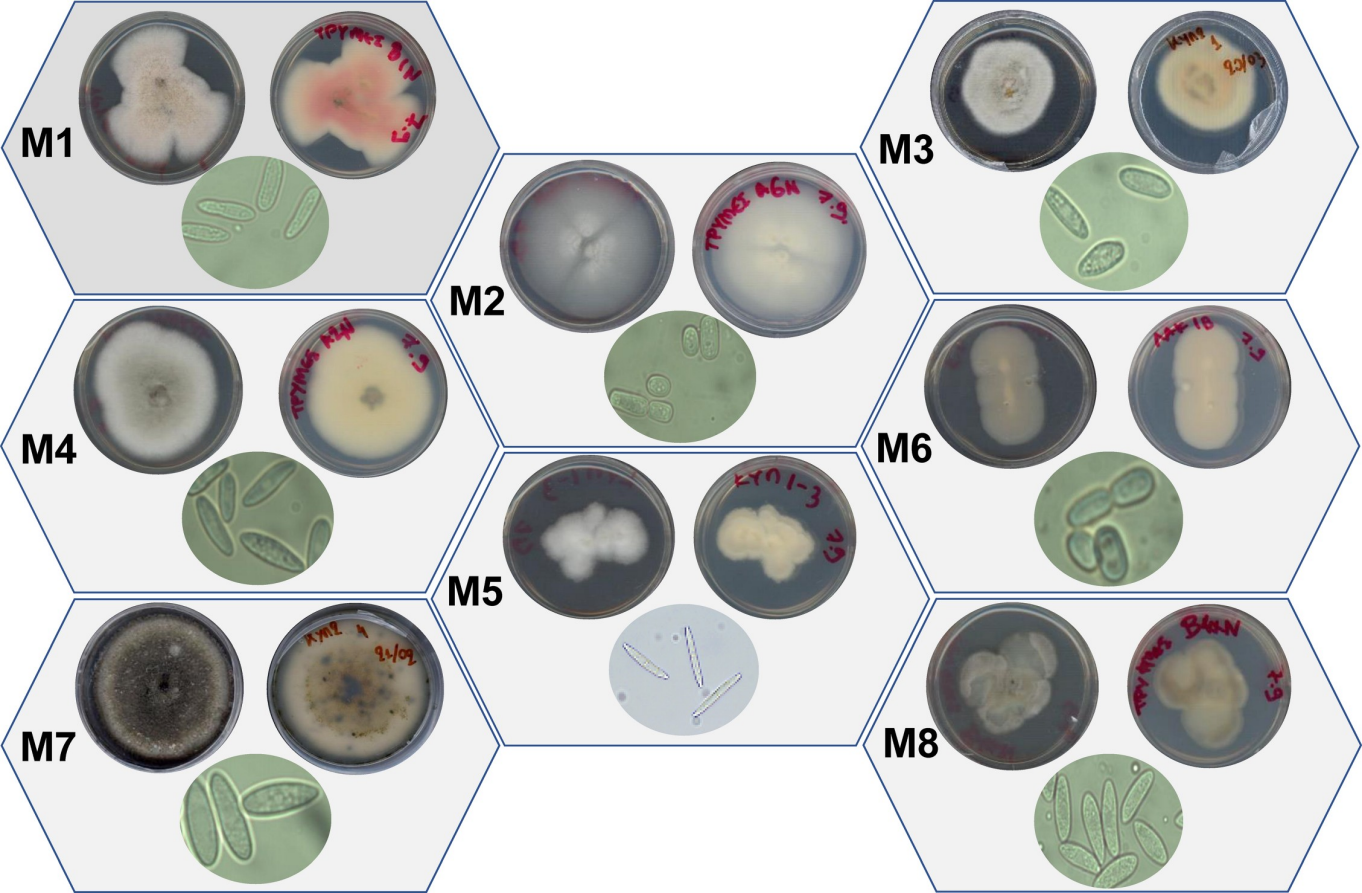
Grouping of the *Colletotrichum acutatum* species complex isolates in morphotypes (M). Eight M based on the phenotypes and the morphology of cultures and spores were identified. Isolates were obtained from infected olive fruits of the cultivar “Koroneiki” from the Peloponnese prefecture of Southern Greece.

Interestingly, the observed phenotypic variation between the cultures of the obtained *C*. *acutatum* species complex isolates grown on PDA, led to their grouping in eight major M; M1-M8 (**Fig 3 and S2 Table**). The morphology and color of the cultures (phenotypes) and the color of the spore masses being produced (conidiomata), where applicable, were the main criteria used in such grouping. More specifically, conidiomata were not formed by the isolates PLS_88 (M2), PLS_110 (M4), PLS_82 (M5), and PLS_81(M6). This, was consistent in the time-course following sub-culturing, and also in new cultures that were started from inocula that had been stored at -80°C (data not shown). Similarly to our results, M of *C*. *acutatum* species complex isolates causing the almond [49] and olive anthracnose [12, 50], have been reported. The grouping of the *C*. *acutatum* isolates in the corresponding M is also important from a plant protection perspective, since the various M could exhibit variable pathogenicity [32, 50] and/or sensitivity to fungicides. Therefore, we have additionally tested the above two hypotheses.

### 3.4. Isolates of different *Colletotrichum acutatum* species complex morphotypes (M) exhibit variable sensitivity to selected a.i. of fungicides

**Fig 4:**
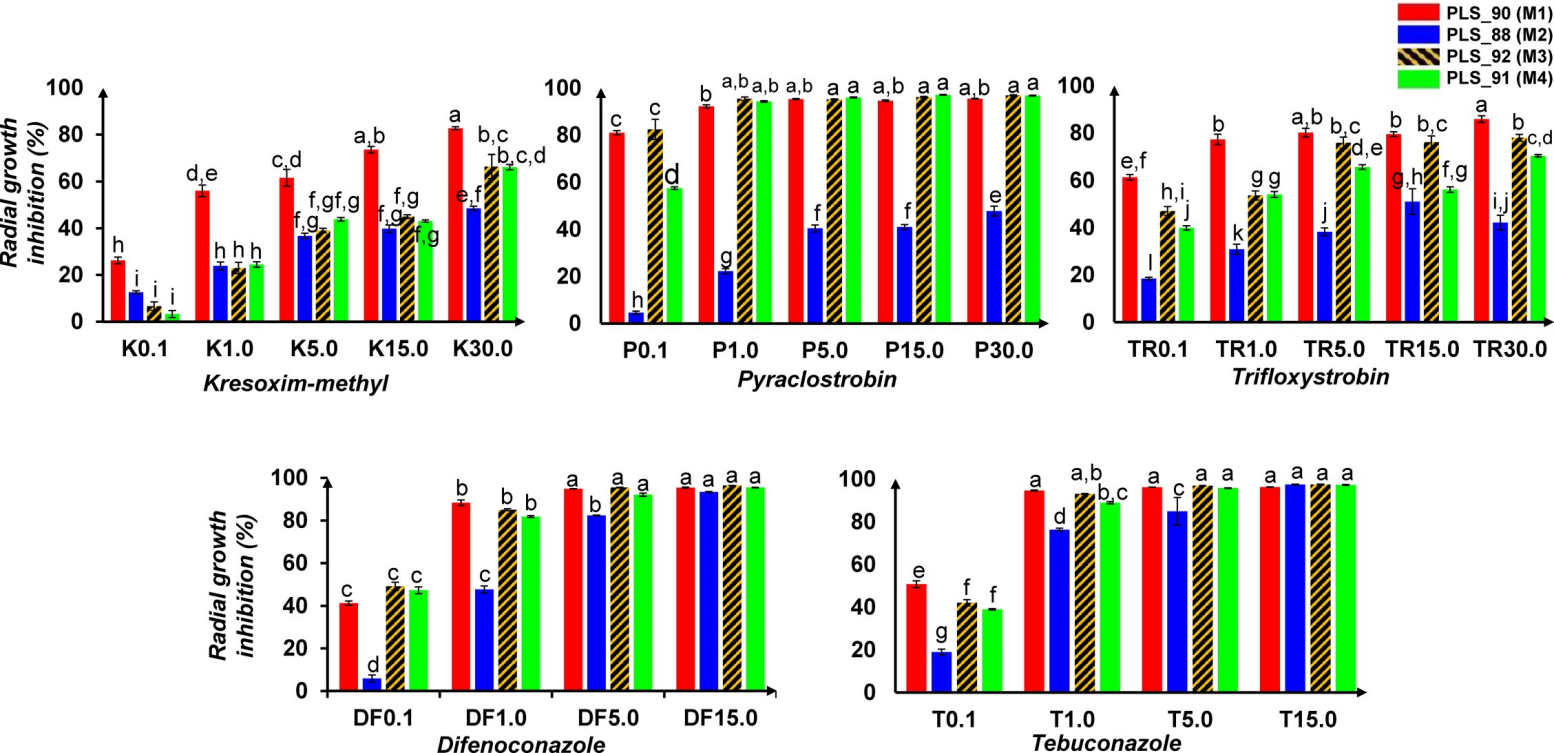
Effects of selected fungicides on the radial growth of *Colletotrichum acutatum* species complex isolates. Strobilurin and triazole fungicides were assessed. The bars correspond to the mean radial growth inhibition (%) of four replications, and the different letters designate statically significant differences performing the Student’s *t*-test (*P* < 95%).

The information on the efficacy of the applied in the olive tree plant protection a.i. to olive pathogenic fungi, is of paramount importance. This becomes even more crucial in the case of the olive anthracnose disease outbreaks in Greece, which result in devastating yield losses. The applied plant protection strategies have been proven ineffective in controlling the disease, which confirms the urge for the acquisition of additional relative knowledge. The information on the efficacy of the applied a.i. in controlling the various genotypes of the fungus, is highly foreseen that will contribute towards combating the issue. Our results, have confirmed the hypothesis that the obtained isolates of different M, exhibit variable sensitivities (**Fig 4**) to major registered a.i. of fungicides (**S1 Table**). Such information, to the best of our knowledge, has not been priory reported. The assessed isolates were selected based on results of preliminary screening on their sensitivity to a.i. (data not shown). The isolates PLS_90 (M1), PLS_88 (M2), PLS_92 (M3), and PLS_91 (M4), were studied.

Results revealed that the isolate PLS_88 (M2) is the least sensitive, whereas the isolate PLS_90 (M1) is the most sensitive to the applied a.i. (**Fig 4**). The isolates PLS_92 (M3) and PLS_91 (M4), exhibit variable sensitivity, with levels ranging between those of the PLS_90 (M1) and PLS_88 (M2). The triazoles difenoconazole and tebuconazole are more effective than the strobilurins being tested, exhibiting high toxicity to all isolates, including the strobilurin-resistant isolate PLS_88 (M2). Among the triazoles, tebuconazole is slightly more toxic, reducing substantially the radial growth of the isolates from the concentration of 1 μg mL^-1^. None of the strobilurins is effective in substantially reducing the growth of the isolate PLS_88 (M2), with trifloxystrobin and kresoxym-methyl being the least effective ones.

Several mechanisms by which fungi acquire resistance to various a.i. have been investigated [51, 52]. Nonetheless, although point mutations at the biochemical target-site [53] have been reported as the main mechanisms by which fungi acquire resistance to major groups of fungicides, including the Qo inhibitors (QoIs) (strobilurins) (FRAC, https://www.frac.info/publications/downloads), the obtained evidence does not allow to conclude on the resistance mechanism of the isolate PLS_88 (M2) to strobilurins, and its slightly reduced sensitivity to triazoles. QoIs is a group of fungicides that exhibits high risk for the development of resistance, with QoIs-resistant fungal isolates appearing just two years following their introduction. In the case of the sterol C_14_-demethylation inhibitors (DMIs, e.g. triazoles), various mechanisms could be responsible for the development of fungal resistance, with DMIs-resistant fungal isolates to appear seven years following their introduction [53].

The above results highlight the necessity of mapping the genotypic variation within *C*. *acutatum* species complex at the level of species/ subspecies, in order to design the appropriate plant protection strategy and rotation of most effective fungicides. For example, knowing that the isolate PLS_88 (M2) prevails in certain orchards, based on our findings, it is evident that applications of strobilurins would have little effect in controlling the disease, whereas in the opposite, they could be effective where the isolates of the M3 and M4 are the predominant ones. Nonetheless, rotation between a.i. is the best agricultural practice for the control of the disease.

### 3.5. The obtained *Colletotrichum acutatum* species complex morphotypes (M) exhibit variable pathogenicity to olive fruits but are similarly virulent to flowers

Based on the results of the assessment of the sensitivity of *C*. *acutatum* species complex isolates to the applied a.i., the isolates PLS_90 (M1) (moderately sensitive to strobilurins and triazoles) and PLS_88 (M2) (resistant to strobilurins), were selected for the study of their pathogenicity to fresh mature fruits and flowers of the cultivars “Koroneiki” and “Kalamon”. The former has been classified as a resistant to the disease cultivar [11]. Additionally, we have tested the hypothesis that the pathogen can infect both healthy fruits with intact, as well as, with mechanically injured epicarp (e.g. pest infestations).

**Fig 5:**
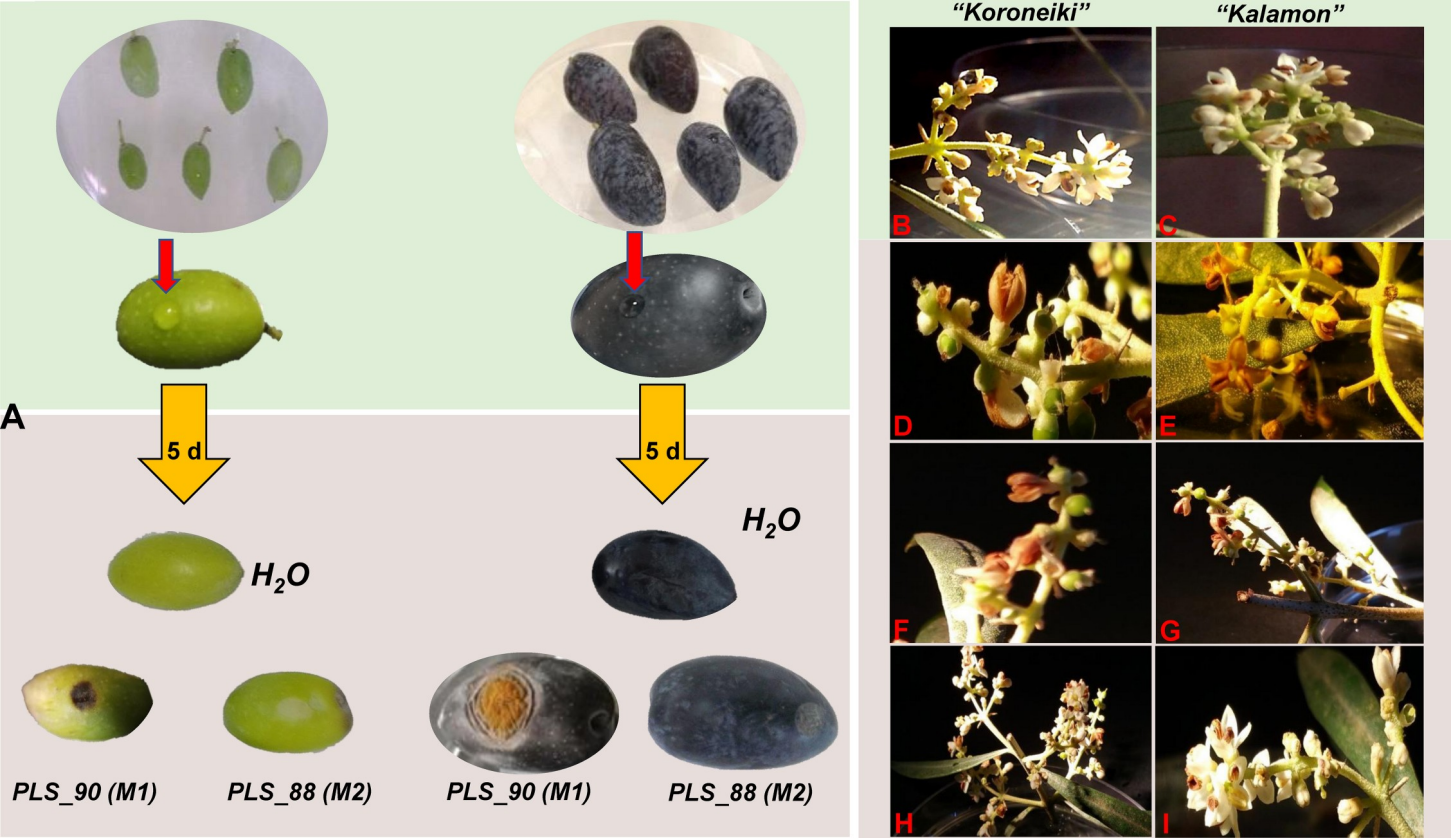
In planta assessment of the pathogenicity of *Colletotrichum acutatum* species complex morphotypes. Olive fruits (A) and flowers (B-I) were artificially infected. Fresh and healthy fruits of the cultivars “Koroneiki” (green) and “Kalamon” (black), were inoculated with spore suspensions (10^6^ spores mL^-1^) of the isolates PLS_88 morphotype (M) 2 (M2), and PLS_90 (M1) (red arrows), without prior injury of their epicarp. Typical symptoms 5 days post-inoculation are depicted following infections with the isolate PLS_90, whereas no symptoms were observed on fruits infected by PLS_88 (A) and the control. Infection of healthy olive flowers (B, C) of the cultivars “Koroneiki” (left column) and “Kalamon” (right column) by spore suspensions (10^6^ spores mL^-1^) of the isolates PLS_88 (D, E) and PLS_90 (F, G), resulted in their infection 2 days post-inoculation. No symptoms were observed on the corresponding control flowers (H, I). Incubation of the inoculated tissues was performed at 22±1°C, in the dark.

Under the conditions set, olive fruits of both cultivars (having intact or not epicarps) that had beeninoculated by *C*. *acutatum*spores,developed the typical symptoms of olive anthracnose five days following their inoculation with the isolate PLS_90 (M1) (**Fig 5A**), which is in line with previous study [14]. Such observation is very important since it confirms the ability of the pathogen to infect healthy fruits, independently of prior pest infestations. Following incubation for additional two weeks, the fruits had been completely colonized by the hyphae of the fungus (**Fig 5A**). The fungi were re-isolated from the infected fruits on PDA and identified as *C*. *acutatum* (data not shown). Interestingly, infection of fruits with the isolate PLS_88 (M2) did not result in the development of symptoms (**Fig 5A**). Although the pathogen could be inoculated from asymptomatic fruits, in some cases that was not feasible. Therefore, here, we cannot conclude whether the absence of symptoms is indicative of the reduced virulence of the isolate or asymptomatic infection [30]. Our findings are in agreement with previous studies on the variable pathogenicity of *Colletotrichum* spp. isolates [32, 50], confirming that the various isolates and M of *C*. *acutatum* species complex exhibit different pathogenicity and virulence. Such observation is very important from a plant protection perspective and confirms results of previous study [12].

Nonetheless, the host recognition and disease development is a complex process, whose outcome is determined by a series of events [54, 55]. Focusing on olive anthracnose, although its pathogenicity is controversial [29], the final outcome depends on many factors, including the combination olive tree cultivar-isolate and fruit maturity. Additionally, the genotypic composition of *C*. *acutatum* isolates regulates their virulence and fitness, and as result, certain genetic groups exhibit superior capacity in adapting to diverse environments and infecting certain hosts. Morphogenetic and physiological differences (e.g. germination of conidia, formation of appressoria), could plausibly explain the differences in their pathogenicity and virulence [50]. Nonetheless, the underlying mechanisms are largely unexplored, therefore, we have applied GC/EI/MS metabolomics in order to mine the endo-metabolomes of the isolated strains and link it to the observed phenotypes.

Furthermore, the inoculation of the flowers of both cultivars by the two isolates was successful; two days following treatments, a slight discoloration appeared at the sites of inoculations and soon after, heavy wilt symptoms were developed (**Fig 5B**). The flowers developed a brown discoloration and hyphae of the fungus were also visible macroscopically two days following inoculation. The fungi were re-isolated from the infected flowers on PDA and identified as *C*. *acutatum* (data not shown). Although the inoculum that was used is substantially higher than what normally occurs in the field, the results confirmed the pathogenicity of both *C*. *acutatum* species complex isolates to olive flowers and their role in the infections of young fruits that occur in the spring and the disease outbreaks. The obtained information should be taken into consideration during the development of plant protection strategies and the decision-making on the appropriate and timely fungicide applications.

### 3.6. Metabolomics analyses of the *Colletotrichum* acutatum species complex morphotypes (M) reveals metabolic differences that could be used in their high-throughput chemotaxonomy and the study of their metabolism in relation to their pathogenicity and sensitivity to fungicides

#### 3.6.1. The GC/EI/MS profiling of the *Colletotrichum acutatum* species complex endo-metabolome can be successfully employed in the chemotaxonomy of its isolates

**Fig 6:**
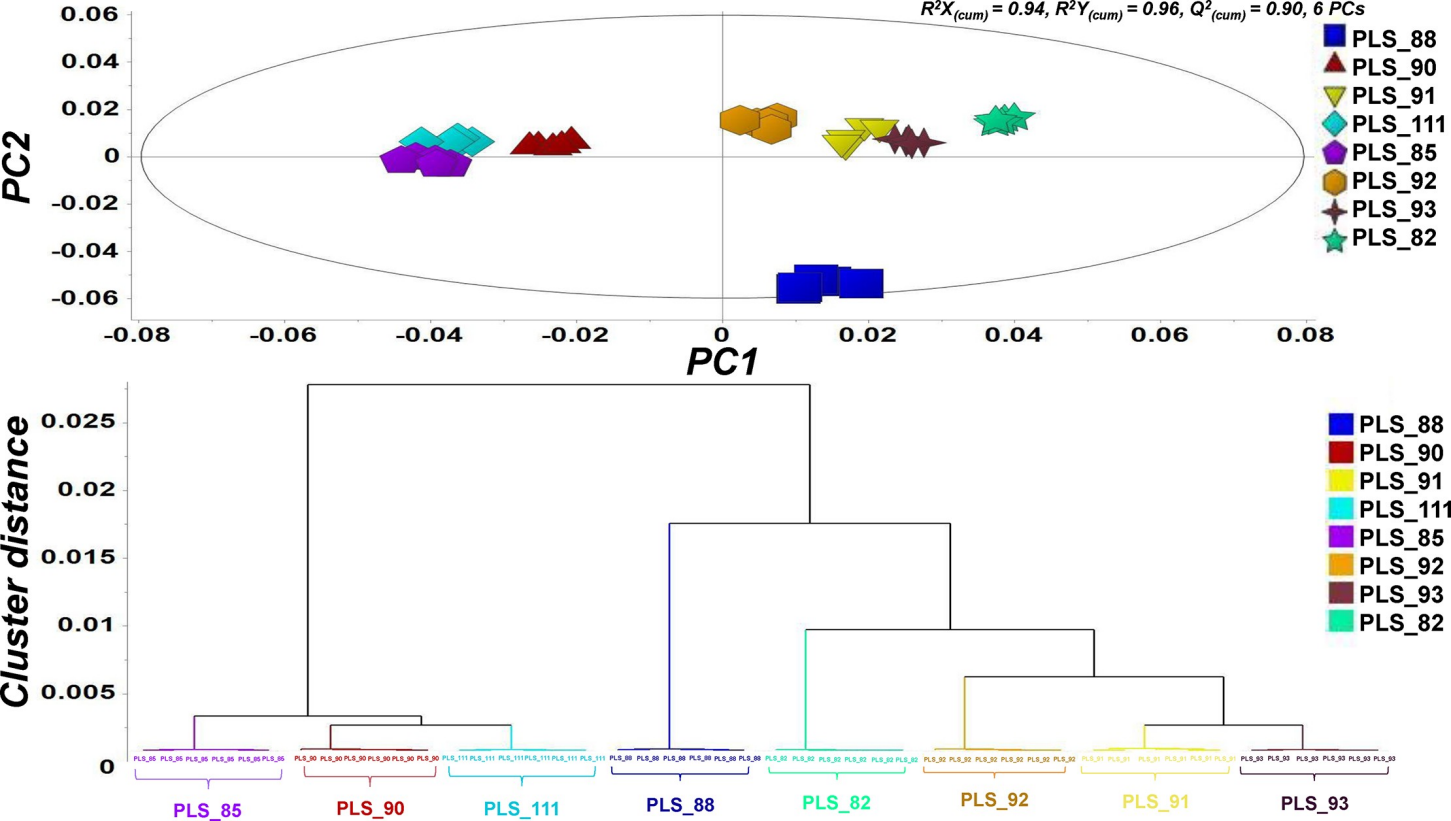
Grouping of *Colletotrichum acutatum* species complex isolates based on the recorded GC/EI/MS metabolite profiles. OPLS-DA score plot for the metabolite profiles of the endo-metabolomes of selected *C*. *acutatum* isolates. The ellipse represents the Hoteling’s T^2^ (95% confidence interval, PC; Principal Component) (A). Corresponding hierarchical cluster analysis (HCA) dendrogram applying the Ward’s linkage method (B). In total, 15 biological replications were performed per isolate, which were finally pooled in groups of three, to finally obtain five pooled samples. Aliquots of each biological replication were combined to obtain quality control (QC) samples [PCs; principal components, *R*^*2*^*X*_*(cum)*_; cumulative fraction of *X* variation, *R*^*2*^*Y*_*(cum)*_; cumulative fraction of *Y* variation, *Q*^*2*^_*(cum)*_; predictive ability].

The applied bioanalytical protocol facilitated the deconvolution of the *Colletotrichum acutatum* species complex isolates’ endo-metabolomes, providing metabolite profiles of high quality, as it is confirmed by the obtained GC/EI/MS total ion chromatograms (TIC) (S2 Fig). The original data set “*Colletotrichum acutatum* (*PMG-06-19*)” in “*.cdf” format, can be freely accessed from the repository of the PMG (https://www.aua.gr/pesticide-metabolomicsgroup/Resources/default.html). The excellent performance of the employed bioanalytical protocol is confirmed by the tight grouping among the biological replications of the different isolates and the lack of outliers (Fig 6).

Results of the GC/EI/MS metabolite profiling, further confirmed the phenotypic grouping of the *C*. *acutatum* species complex isolates in the various M; the corresponding obtained metabolite profiles were distinct, forming separate groups, with isolates belonging to the same M clustered close together (**Fig 6**). The robustness of the developed model is confirmed by the parameters of cross validation for 6 principal components (PCs); cumulative fraction of *X* variation, *R*^*2*^*X*_*(cum)*_ = 0.94, cumulative fraction of *Y* variation, *R*^*2*^*Y*_*(cum)*_ = 0.96, and predictive ability, *Q*^*2*^_*(cum)*_ = 0.90.

*Colletotrichum* spp. Is a highly complex genus, with a proposed number of species ranging from 29 to over 700, causing severe diseases to a wide range of hosts [27, 28]. Although there has been an effort towards the improvement of its taxonomy, to date, it still remains inconclusive, with DNA sequencing being the main employed technology [27, 48]. Our data suggest that the applied GC/EI/MS-based chemotaxonomy approach could serve as an additional tool, complementing the already applied, towards the elucidation of the taxonomy of *C*. *acutatum* isolates, and possibly that of the *Colletotrichum* spp. The applicability of chemotaxonomy in the taxonomy of *Colletotrichum* spp. Is in accordance with previous studies on filamentous fungi such as, *Rhizoctonia solani* [35], and *Alternaria* spp., *Aspergillus* spp., *Fusarium* sp. And *Penicillium* spp. [36], further confirming its applicability and potential. Furthermore, such approach could provide information on physiological parameters such as, pathogenicity and responses to stresses [35] that could be further exploited within the context of plant protection, linking the genotypes with the obtained phenotypes [56].

Interestingly, the isolate PLS_88 (M2), which exhibits lower sensitivities to the applied a.i. and does not cause visible symptoms in olive fruits following their inoculation with spore suspensions, is clustered far from the rest isolates (**Fig 6**). Based on such observation, we have undergone the task of further mining the differences in its endo-metabolome compared to the rest analyzed isolates, in order to gain insights into the biochemical basis of its differential behavior and phenotypes.

#### 3.6.2. The altered metabolite profile of the isolate PLS_88 of the *Colletotrichum acutatum*, morphotype 2 (M2) confirms its reduced sensitivity to fungicides and its differential pathogenicity

GC/EI/MS analyses revealed that the isolate PLS_88 (M2) of *Colletotrichum acutatum*, which is resistant to strobilurins and exhibits decreased pathogenicity to olive fruits, has a distinct metabolite profile (Fig 6). Therefore, we have further investigated whether such features could be, at least partially, attributed to its unique metabolite composition. Nonetheless, the comprehensive interpretation of the results of metabolomics was beyond the aim of the present research.

**Fig 7:**
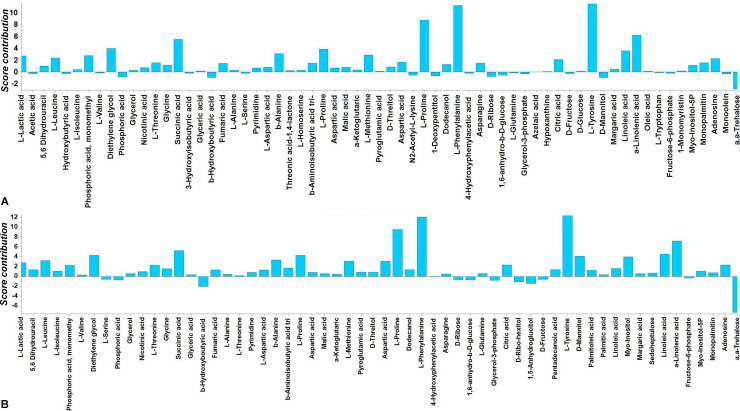
Differences between the GC/EI/MS metabolite profiles of selected *Colletotrichum acutatum* species complex isolates. Contribution plot displaying metabolite differences between the endo-metabolome of the *Colletotrichum acutatum* isolate PLS_88 morphotype (M) 2 (M2) (in scale units), and that of the average of the rest M (A), or the isolate PLS_90 (M1) (B). High absolute values correspond to metabolites with the highest leverage on the observed discrimination. Positive values correspond to metabolites with increased levels in the isolate PLS_88 (M2), while negative to those with decreased levels compared to the rest isolates.

**Fig 8:**
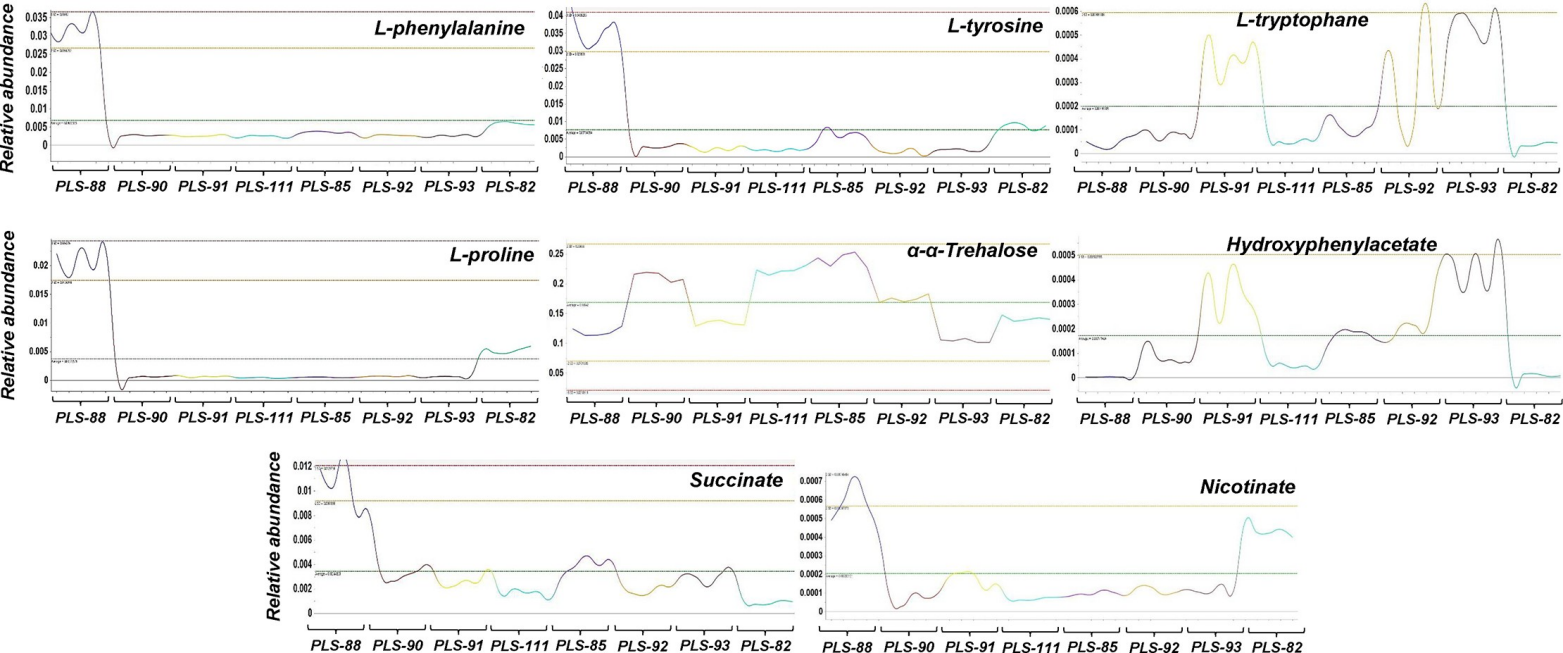
Fluctuations in the content of the signatory metabolites of the analyzed *Colletotrichum acutatum* species complex isolates. The fluctuation of the relative abundance of selected metabolites-biomarkers whose levels differ substantially, and additionally play important roles in fungal metabolism, pathogenesis, and stress responses, are displayed.

In the obtained contribution plot, in which differences between the endo-metabolomes of the isolate PLS_88 (M2) and those of the rest isolates are displayed, it is evident that the levels of various metabolites differ substantially (**Fig 7A**). Among the most important signatory metabolites of the isolate PLS_88 (M2) were, hydroxyphenylacetate, myo-inositol-P, pentadecanoate, nicotinate, citrate, adenosine, *β*-alanine, methionine, fumarate, lactate, glycine, linoleic acid, succinate, mannitol, aspartate, *α*-linolenic, L-alanine, L-tyrosine, L-phenylalanine, L-proline, and *α*,*α*-trehalose. The fluctuations in the content of selected *C*. *acutatum* species complex metabolites that play key roles in fungal physiology are displayed in **Fig 8**. Additionally, the pairwise comparison between the isolates PLS_88 (M2) and PLS_90 (M1) revealed the differences between their endo-metabolomes (**Fig 7B and S3 Fig**). The two isolates were selected for such comparison since their pathogenicity and sensitivity to a.i. were also assessed.

Hydroxyphenylacetate, *α*,*α*-trehalose, and L-proline, are metabolites for which there is solid evidence in the literature in support of their involvement in fungal pathogenicity and responses to stresses, as described below. Therefore, it is plausible to suggest a similar role for these metabolites in the corresponding functions of the *C*. *acutatum* isolates that were investigated.

Phenylacetic acid and its derivatives, including hydroxyphenylacetate, are phytotoxic fungal metabolites produced by various species such as, *R*. *solani* [35, 57] and *Streptomyces humidus* [58]. They exhibit antimicrobial properties [58, 59] and interestingly, a strong correlation seems to exist between the capacity of fungi to synthesize phenylacetic acid and its derivatives, and their pathogenicity [60]. Such observation is in complete agreement with our observations, supporting the hypothesis that the hydroxyphenylacetate-producing capacity of the *C*. *acutatum* isolates that were studied, exhibits a strong positive correlation to their pathogenicity to olive fruits and the development of the corresponding symptoms. Nonetheless, a large-scale study is further required, including the study of pathogenesis and metabolism of a large number of isolates and olive cultivars to further confirm and validate our hypotheses.

On the other hand, a negative correlation between the content of *C*. *acutatum* hyphae in *α*,*α*-trehalose and their sensitivity to the applied a.i. was recorded, which is in agreement with previous study on the resistance of *Fusarium graminearum* strains to the a.i. carbendazim [61]. *α*,*α*-Trehalose is a non-reducing disaccharide exhibiting unique properties and serves as an energy source, and additionally protects the structure and function of cell membranes, regulates stress tolerance (e.g. desiccation, freezing, starvation), sporulation, glycolysis, and pathogenicity [62–66]. Focusing on the latter, there is increasing evidence that suggest its role in the regulation of virulence traits in various species [67–69]. In *Magnaporthe grisea*, mutation of the *OtsA* gene reduces substantially its pathogenicity plausibly due to the interference with the penetration of the hyphae into the plant tissues [69]. Although our data cannot prove the role of *α*,*α*-trehalose in the pathogenesis of *C*. *acutatum*, based on the above information, it is likely that low levels of the disaccharide affects negatively its pathogenicity.

Furthermore, although fungal responses to abiotic stresses are under the control of a complex regulatory mechanism, the levels of metabolites such as L-proline, could serve as a reliable indicator of the stress level and metabolite responses of organisms to stresses (e.g. oxidative, osmotic) [70–72]. It is a reacting oxygen species (ROS)-scavenger, osmoregulator, and additionally stabilizes proteins and the cell membranes [70, 72]. Its increased levels have been reported among the responses of fungi to treatments with fungicides [61]. Our findings are in agreement with the latter study, indicating the positive correlation between the levels of L-proline and the resistance of *C*. *acutatum* isolates to the applied a.i.

Finally, the increased levels of the isolate PLS_88 (M2) in the aromatic amino acids L-phenylalanine and L-tyrosine, were among the most significant findings. The results are in agreement with previous study on *F*. *graminearum*, in which resistant to carbendazim strains had high levels of aromatic amino acids [61]. They are cornerstones in fungal metabolism, being precursors of the numerous secondary metabolites that are synthesized via the indole alkaloid, phenylpropanoid, tropane, piperidine and pyridine alkaloid biosynthetic pathways. It seems that their activation, leads to the biosynthesis of secondary fungal metabolites, which possibly assists the pathogen to overcome the stress caused by the applied a.i.

## Conclusions

The results of our research revealed that *C*. *acutatum* species complex is the predominant cause of the outbreaks of olive anthracnose in Southern Greece, with several of its obtained isolates to be assigned to various M based on their phenotypes. Differential levels of pathogenicity and sensitivity to the applied a.i. were observed for the M being assessed. Among the major findings is that certain M exhibit resistance to the a.i. being tested, making necessary the rotation between copper-based fungicides, strobilurins, and triazoles. The findings dictate the need for the comprehensive mapping of the geographical distribution of *C*. *acutatum*, in order to design customized plant protection strategies and avoid issues such as the development of resistant genotypes. The rotation of a.i., combined with agricultural practices such as, pruning of olive trees, systematic removal and destruction of the remaining infected fruits, early harvesting, and regular monitoring of the environmental conditions, is highly foreseen that will help towards minimizing yield losses in a time course. Additionally, metabolomics proved to be an effective tool in the routine high-throughput chemotaxonomy of *C*. *acutatum*, and the in-depth study of their metabolism in relation to their pathogenicity and sensitivity to fungicides. Sets of the discovered biomarkers could be used in the high-throughput assessment of the pathogenicity of *C*. *acutatum* isolates and their sensitivity. Such integrative approach could be adapted by olive producing countries towards minimizing the losses caused by olive anthracnose.

## Supporting information

S1 Fig Samples of olive fruits of the variety “Koroneiki” exhibiting various symptoms consistent with fungal infections (A), and the corresponding locations of sampling from the Peloponnese prefecture in Southern Greece (B).
(TIFF)

S2 Fig Representative total ion chromatograms (TIC) of the endo-metabolomes of the *Colletotrichum acutatum* species complex isolates PLS_88 (M1) and PLS_90 (M2). Annotations for representative identified metabolites are displayed.
(TIFF)

S3 Fig OPLS-DA score plot for the GC/EI/MS metabolite profiles of the endo-metabolomes of the *Colletotrichum acutatum* species complex isolates PLS_88 (M1) and PLS_90 (M2). The ellipse represents the Hoteling's T^2^ at a 95% confidence interval (PC; Principal Component). In total, 15 biological replications were performed per isolate, which were finally pooled in groups of three, to finally obtain five pooled samples. Aliquots of each biological replication were combined to obtain quality control (QC) samples [PCs; principal components, *R*^*2*^*X*_*(cum)*_; cumulative fraction of *X* variation, *R*^*2*^*Y*_*(cum)*_; cumulative fraction of *Y* variation, *Q*^*2*^_*(cum)*_; predictive ability].
(TIFF)

S1 Table Registered uses of active ingredients (a.i.) of fungicides in the plant protection of olive tree in Greece (source: Ministry of rural development and food, access: 10/2019). (DOCX)

S2 Table Grouping of the selected *Colletotrichum acutatum* species complex isolates in morphotypes (M). (DOCX)

S3 Table Unidentified isolates that were not consistent with features of *Colletotrichum* sp. and were excluded from further investigation. (DOCX)

S1 File (DOCX)

S1 Raw Images (PDF)
