# Supplementary figures and images for: An integrated approach to improve plant protection against olive anthracnose caused by the Colletotrichum acutatum species complex
Source: PLoS One. 2020 May 29;15(5):e0233916. doi: 10.1371/journal.pone.0233916 (PMC7259717; doi:10.1371/journal.pone.0233916)

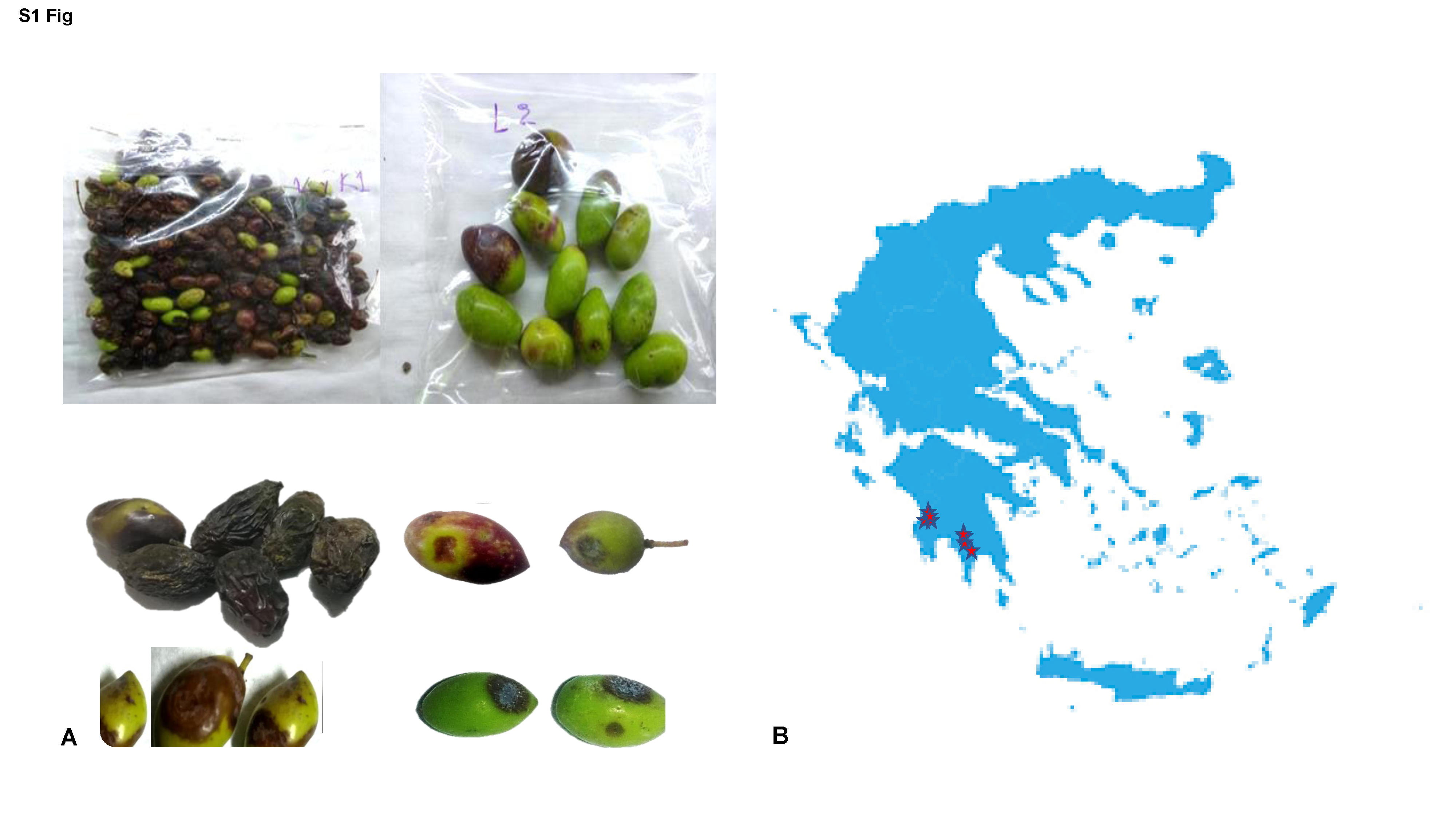

Supplement: S1 Fig — Samples of olive fruits of the variety “Koroneiki” exhibiting various symptoms consistent with fungal infections (A), and the corresponding locations of sampling from the Peloponnese prefecture in Southern Greece (B). (TIFF) [file pone.0233916.s001.tiff]

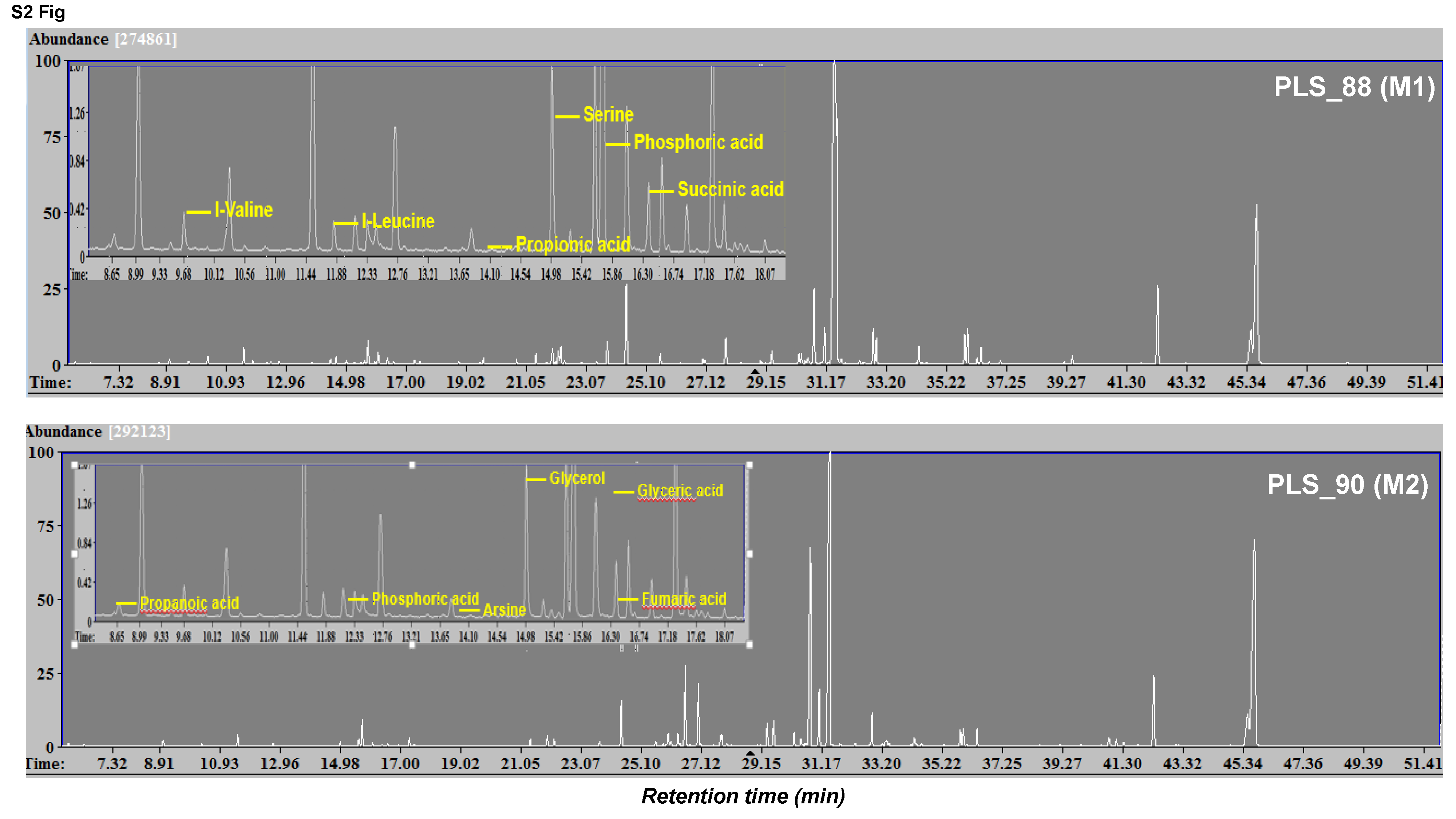

Supplement: S2 Fig — Annotations for representative identified metabolites are displayed. (TIFF) [file pone.0233916.s002.tiff]

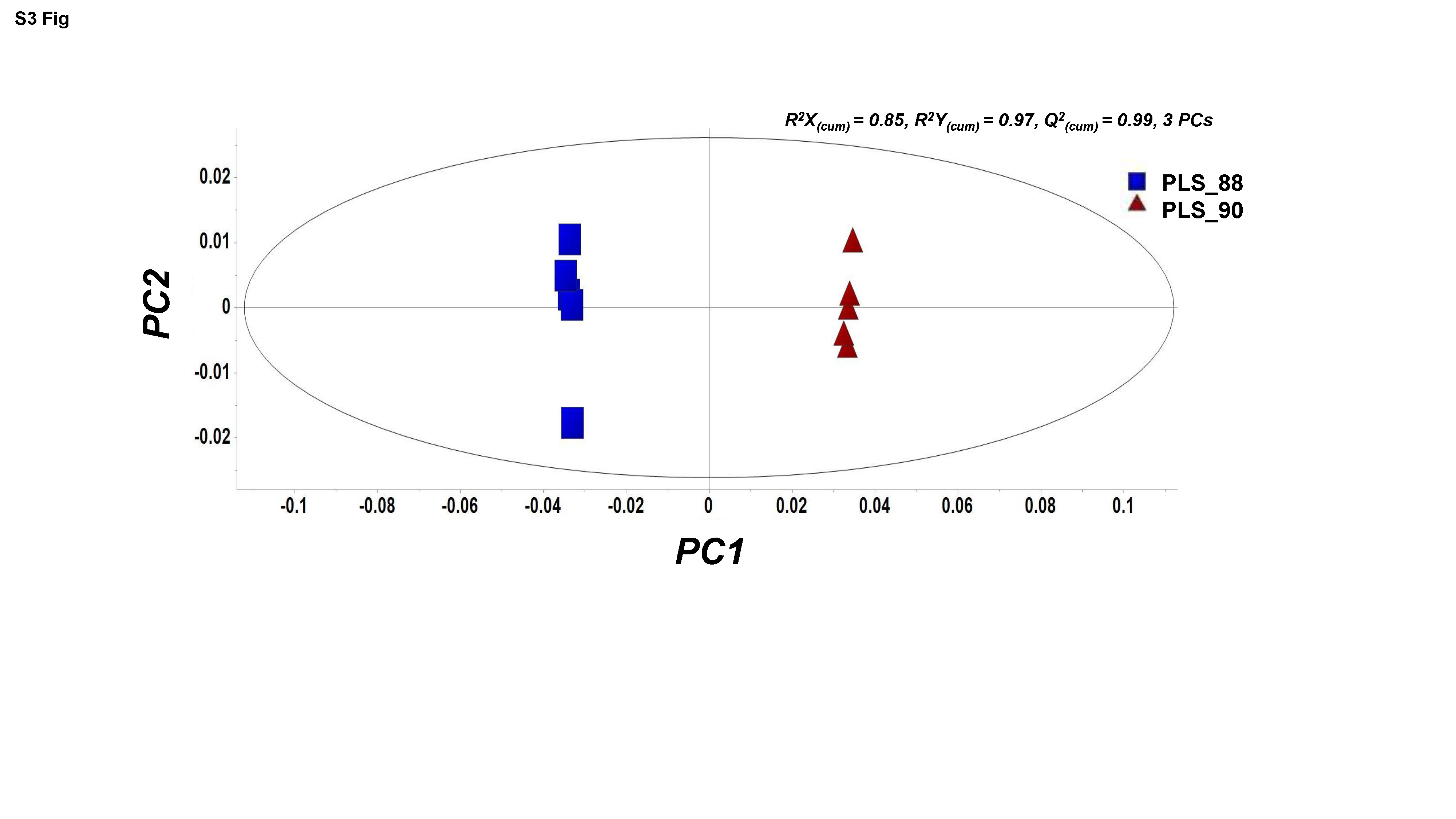

Supplement: S3 Fig — The ellipse represents the Hoteling's T2 at a 95% confidence interval (PC; Principal Component). In total, 15 biological replications were performed per isolate, which were finally pooled in groups of three, to finally obtain five pooled samples. Aliquots of each biological replication were combined to obtain quality control (QC) samples [PCs; principal components, R2X(cum); cumulative fraction of X variation, R2Y(cum); cumulative fraction of Y variation, Q2(cum); predictive ability]. (TIFF) [file pone.0233916.s003.tiff]
