# Supplementary material for: An integrated approach to improve plant protection against olive anthracnose caused by the Colletotrichum acutatum species complex
Source: PLoS One. 2020 May 29;15(5):e0233916. doi: 10.1371/journal.pone.0233916 (PMC7259717; doi:10.1371/journal.pone.0233916)
Supplement: S1 Table — (DOCX) [file pone.0233916.s004.docx]

| **A.I.** | **Olive anthracnose (*Colletotrichum* sp.)** | **Cladosporium rot (*Cladosporium* sp.)** | **Peacock spot (*Spilocaea oleagina*)** | **Maximum applications per growing season** | **Pre-harvests interval (days)** |
| --- | --- | --- | --- | --- | --- |
| **Azoxystrobin** |  |  |  | 1 | During flowering |
| [**Bordeaux mixture**](http://wwww.minagric.gr/syspest/syspest_Detail_drastiko.aspx?onomadron=Bordeaux%20mixture) |  |  |  | 2 | 21 |
| [**Copper hydroxide**](http://wwww.minagric.gr/syspest/syspest_Detail_drastiko.aspx?onomadron=Copper%20hydroxide) |  |  |  | 2 | 21 |
| [**Copper oxide**](http://wwww.minagric.gr/syspest/syspest_Detail_drastiko.aspx?onomadron=Copper%20oxide) |  |  |  | 2 | 21 |
| [**Copper oxychloride**](http://wwww.minagric.gr/syspest/syspest_Detail_drastiko.aspx?onomadron=Copper%20oxychloride) |  |  |  | 2 | 21 |
| **Difenoconazole** |  |  |  | 2/14days | 30 |
| **Dodine** |  |  |  | 2/7days | 7 |
| **Fenbuconazole** |  |  |  | 1 | During flowering |
| **Kresoxim-methyl** |  |  |  | 1-2 | 30 |
| **Mancozeb** |  |  |  | 1-3 | 21 |
| **Pyraclostrobin** |  |  |  | Oil varieties :  2/21days  Table varieties :  3/21days | 105 |
| **Tebuconazole** |  |  |  | 1 | Before flowering |
| [**Tribasic copper sulfate**](http://wwww.minagric.gr/syspest/syspest_Detail_drastiko.aspx?onomadron=Tribasic%20copper%20sulfate) |  |  |  | 2 | 21 |
| **Trifloxystrobin** |  |  |  | 1-2 | 14 |

**S1 Table.** **Registered uses of active ingredients (a.i.) of fungicides in the plant protection of olive tree in Greece (source: Ministry of Rural Development and Food, access: 10/2019)**
