# Supplementary material for: An integrated approach to improve plant protection against olive anthracnose caused by the Colletotrichum acutatum species complex
Source: PLoS One. 2020 May 29;15(5):e0233916. doi: 10.1371/journal.pone.0233916 (PMC7259717; doi:10.1371/journal.pone.0233916)
Supplement: S2 Table — (DOCX) [file pone.0233916.s005.docx]

**S2 Table. Grouping of the selected *Colletotrichum acutatum* species complex isolates in morphotypes (M)**

| **Morphotypes (M)** | ***Colletotrichum acutatum.* species complex strains** | | **Location** | | **Variety** | **Date** | **Spore dimensions (μm)** | **Spore shape** |
| --- | --- | --- | --- | --- | --- | --- | --- | --- |
| Μ1 | | PLS_86  PLS_90  PLS_111 | | Lakonia  Lakonia  Lakonia | Koroneiki  Koroneiki  Koroneiki | 2016-‘17  2016-‘17  2017-‘18 | 12.38 ± 0.95 x 4.25 ± 0.50  11.75 ± 0.50 x 4.75 ± 0.50  11.50 ± 0.75 x 4.35 ± 0.50 | Fusiform  Fusiform  Fusiform |
| Μ2 | | PLS_87  PLS_88 | | Lakonia  Messinia | Koroneiki  Koroneiki | 2016-‘17  2016-‘17 | 8.25 ± 0.95 x 5.00 ± 0.81  8.25 ± 1.25 x 4.75 ± 0.95 | Elliptical  Elliptical |
| M3 | | PLS_92  PLS_109  PLS_112 | | LakoniaMessinia  Messinia | Koroneiki  Koroneiki  Koroneiki | 2016-‘17  2017-‘18  2017-‘18 | 12.75 ± 0.50 x 3.75 ± 0.50  11.65 ± 0.50 x 4.50 ± 0.25  12.00 ± 0.45 x 4.25 ± 0.35 | Cylindrical  Cylindrical  Cylindrical |
| Μ4 | | PLS_84  PLS_91  PLS_93  PLS_110 | | Lakonia  Messinia  Messinia  Messinia | Koroneiki  Koroneiki  Koroneiki  Koroneiki | 2016-‘17  2016-‘17  2017-‘18  2017-‘18 | 13.50 ± 1.73 x 3.50 ± 0.57  15.25 ± 0.95 x 4.75 ± 0.50  12.75 ± 0.50 x 3.75 ± 0.50  13.00± 0.25 x 3.95 ± 0.30 | Cylindrical  Cylindrical  Cylindrical  Cylindrical |
| M5 | | PLS_82 | | Lakonia | Koroneiki | 2016-‘17 | 14.00 ± 0.81 x 2.00± 0.10 | Elliptical |
| M6 | | PLS_81 | | Messinia | Koroneiki | 2016-‘17 | 9.00 ± 0.35 x 4.10 ± 0.20 | Elliptical |
| M7 | | PLS_83  PLS_102 | | Messinia  Lakonia | Koroneiki  Koroneiki | 2016-‘17  2017-‘18 | 11.75 ± 1.50 x 4.12 ± 0.25  14.75 ± 0.95 x 5.12 ± 0.62 | Cylindrical  Cylindrical |
| M8 | | PLS_85 | | Lakonia | Koroneiki | 2016-‘17 | 12.75 ± 2.36 x 4.25 ± 0.50 | Cylindrical |
