# Supplementary material for: An integrated approach to improve plant protection against olive anthracnose caused by the Colletotrichum acutatum species complex
Source: PLoS One. 2020 May 29;15(5):e0233916. doi: 10.1371/journal.pone.0233916 (PMC7259717; doi:10.1371/journal.pone.0233916)
Supplement: S3 Table — (DOCX) [file pone.0233916.s006.docx]

**S3 Table. Unidentified isolates that were not consistent with features of *Colletotrichum* sp. and were excluded from further investigation**.

|  | **Isolate’s code** | **Location** | **Variety** | **Date of isolation** |
| --- | --- | --- | --- | --- |
|  | PLS_100 | Messinia | Koroneiki | 2017-‘18 |
|  | PLS_101 | Messinia | Koroneiki | 2017-‘18 |
|  | PLS_103 | Messinia | Koroneiki | 2017-‘18 |
|  | PLS_104 | Messinia | Koroneiki | 2017-‘18 |
|  | PLS_105 | Messinia | Koroneiki | 2017-‘18 |
|  | PLS_106 | Messinia | Koroneiki | 2017-‘18 |
|  | PLS_107 | Messinia | Koroneiki | 2017-‘18 |
|  | PLS_108 | Messinia | Koroneiki | 2017-‘18 |
|  | PLS_110 | Messinia | Koroneiki | 2017-‘18 |
|  | PLS_113 | Messinia | Koroneiki | 2017-‘18 |
|  | PLS_114 | Lakonia | Koroneiki | 2017-‘18 |
|  | PLS_115 | Messinia | Koroneiki | 2017-‘18 |
|  | PLS_199 | Lakonia | Koroneiki | 2016-‘17 |
|  | PLS_94 | Lakonia | Koroneiki | 2016-‘17 |
|  | PLS_95 | Lakonia | Koroneiki | 2016-‘17 |
|  | PLS_96 | Lakonia | Koroneiki | 2016-‘17 |
|  | PLS_97 | Lakonia | Koroneiki | 2016-‘17 |
|  | PLS_98 | Lakonia | Koroneiki | 2016-‘17 |
|  | PLS_99 | Messinia | Koroneiki | 2016-‘17 |
|  | PLS_198 | Messinia | Koroneiki | 2016-‘17 |
