# Supplementary material for: An integrated approach to improve plant protection against olive anthracnose caused by the Colletotrichum acutatum species complex
Source: PLoS One. 2020 May 29;15(5):e0233916. doi: 10.1371/journal.pone.0233916 (PMC7259717; doi:10.1371/journal.pone.0233916)
Supplement: S1 File — (DOCX) [file pone.0233916.s007.docx]

**An integrated approach to investigate olive anthracnose caused by *Colletotrichum acutatum* *sensu stricto*: isolation, assessment of pathogenicity and sensitivity to fungicides, and metabolomics analyses**

**Stefanos Kolainis.^1*^, Koletti Anastasia^1*^, Lykogianni Maira^1,2*^, Karamanou Dimitra^1^, Danai Gkizi^3^, Sotirios E. Tjamos^3^, Antonios Paraskeuopoulos^4^, Konstantinos A. Aliferis^1,5^**

# Supplementary Materials and Methods

**Extraction of *Colletotrichum acutatum* hyphae for GC/EI/MS metabolomics analyses**

The extraction of the hyphae was performed using a mixture (0.5 mL) of methanol-ethyl acetate (1:1, v/v) [1]. Sonication followed in an ultrasonic bath (Branson 1210, Connecticut, USA) for 20 min and stirring for 2h in an orbital shaker (GFL 3006, Burgwedel, Germany). Then, the crude extracts were filtered through PTFE filters (0.2 μm, Macherey-Nagel, Duren, Germany) for the removal of debris and a solution of ribitol (Sigma-Aldrich Ltd, Steinheim, Germany) (20 μL of 0.2 mg mL^-1^ in methanol) was added as the internal standard. Finally, the extracts were evaporated using a vacuum concentrator (Labconco, Kansas City, MO, USA).

**Derivatization of *Colletotrichum acutatum* extracts**

The derivatization of the dried extracts was performed in a two-step process [1]; initially, for methoxymation, a methoxylamine hydrochloride solution in pyridine (80 μL, 20 mg mL^-1^) was added in the dried extracts, followed by incubation for 2 h at 30°C, in a water bath. For silylation, MSTFA (80 μL) was then added, and the solutions were incubated at 37°C for 1.5 h. The obtained derivatized samples were added into microinserters (180 μL, Macherey-Nagel) in glass autosampler vials (2 mL, Macherey-Nagel). Experimental blanks were additionally prepared for the detection of metabolic features not related to the analyzed biological material (e.g. contamination of the analytical platform, column bleeding).

# Settings of gas chromatography/electron impact/mass spectrometry (GC/EI/MS) metabolomics analyses

Previously described settings were used in the analyses [1, 2]. The analyzer was operating in positive electron ionization (70 eV) and full scanning spectra were acquired in the mass range 50-800 Da, in a rate of 4 scans sec^-1^. An initial delay of 10 min was applied. The temperatures were: MS source; 230°C, quadruple; 150°C, injector; 230°C. The samples 1 μL were injected on column at a split ratio of 5:1. Helium was used as the carrier gas at a flow rate of 1 mL min^-1^. The program of the oven was; 70°C for 5 min, increased 5°C min^-1^ to 310°C, kept for 1 min.

# References

1. Kalampokis IF, Kapetanakis GC, Aliferis KA, Diallinas G. Multiple nucleobase transporters contribute to boscalid sensitivity in Aspergillus nidulans. Fungal Genet Biol. 2018; 115:52-63.

2. Kostopoulou S, Ntatsi G, Arapis G, Aliferis KA. Assessment of the effects of metribuzin, glyphosate, and their mixtures on the metabolism of the model plant Lemna minor L. applying metabolomics. Chemosphere. 2020; 239:124582.
