# Supplementary material for: An integrated approach to improve plant protection against olive anthracnose caused by the Colletotrichum acutatum species complex
Source: PLoS One. 2020 May 29;15(5):e0233916. doi: 10.1371/journal.pone.0233916 (PMC7259717; doi:10.1371/journal.pone.0233916)
Supplement: S1 Raw Images — (PDF) [file pone.0233916.s008.pdf]

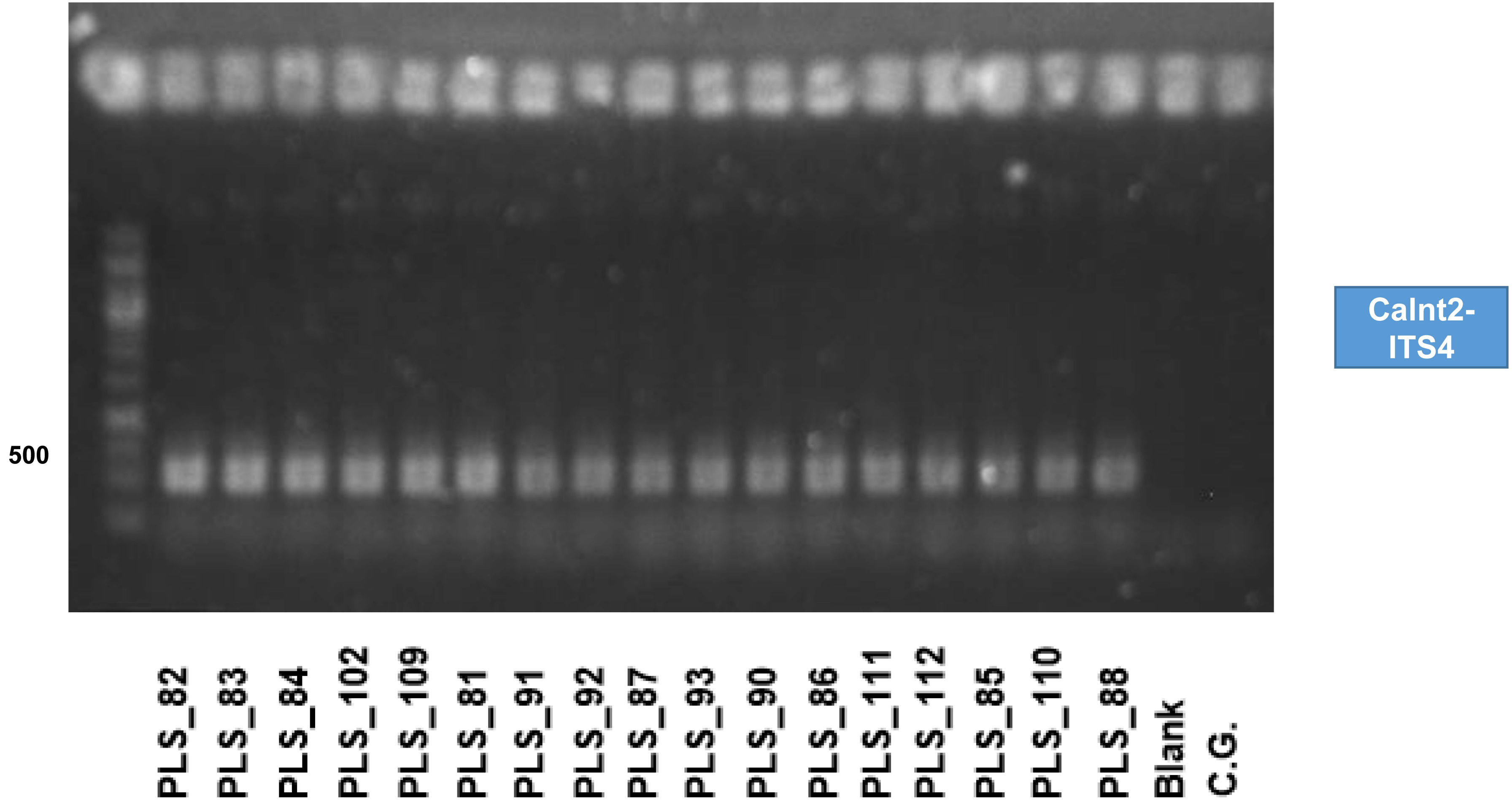

PCR products
1% agarose gel
110V, 30’
150ng template DNA
Reaction volume 50µl
Thermo scientific PCR kit

Fig 2A

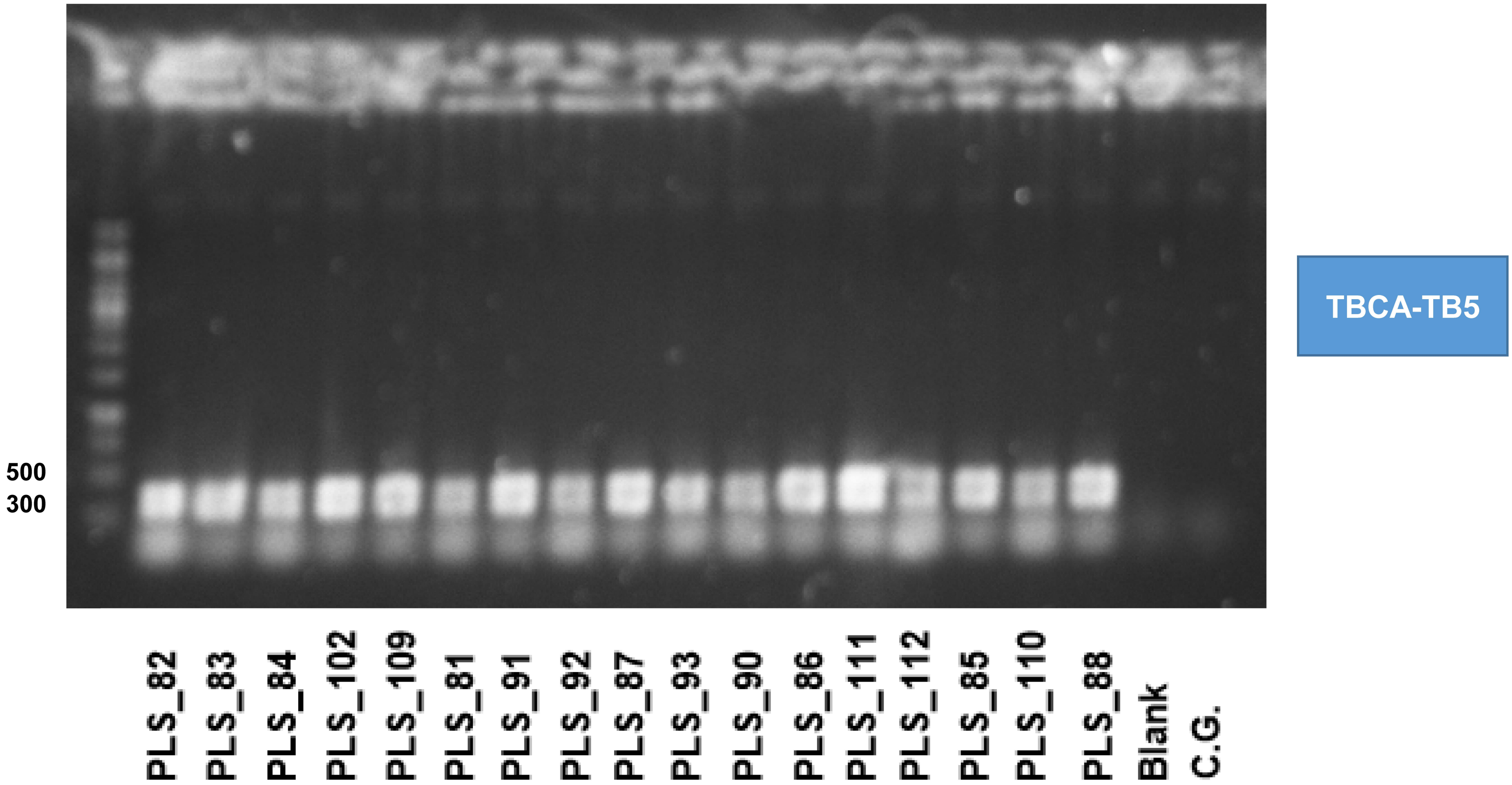

PCR products
1% agarose gel
110V, 30’
150ng template DNA
Reaction volume 50µl
Thermo scientific PCR kit

Fig 2B

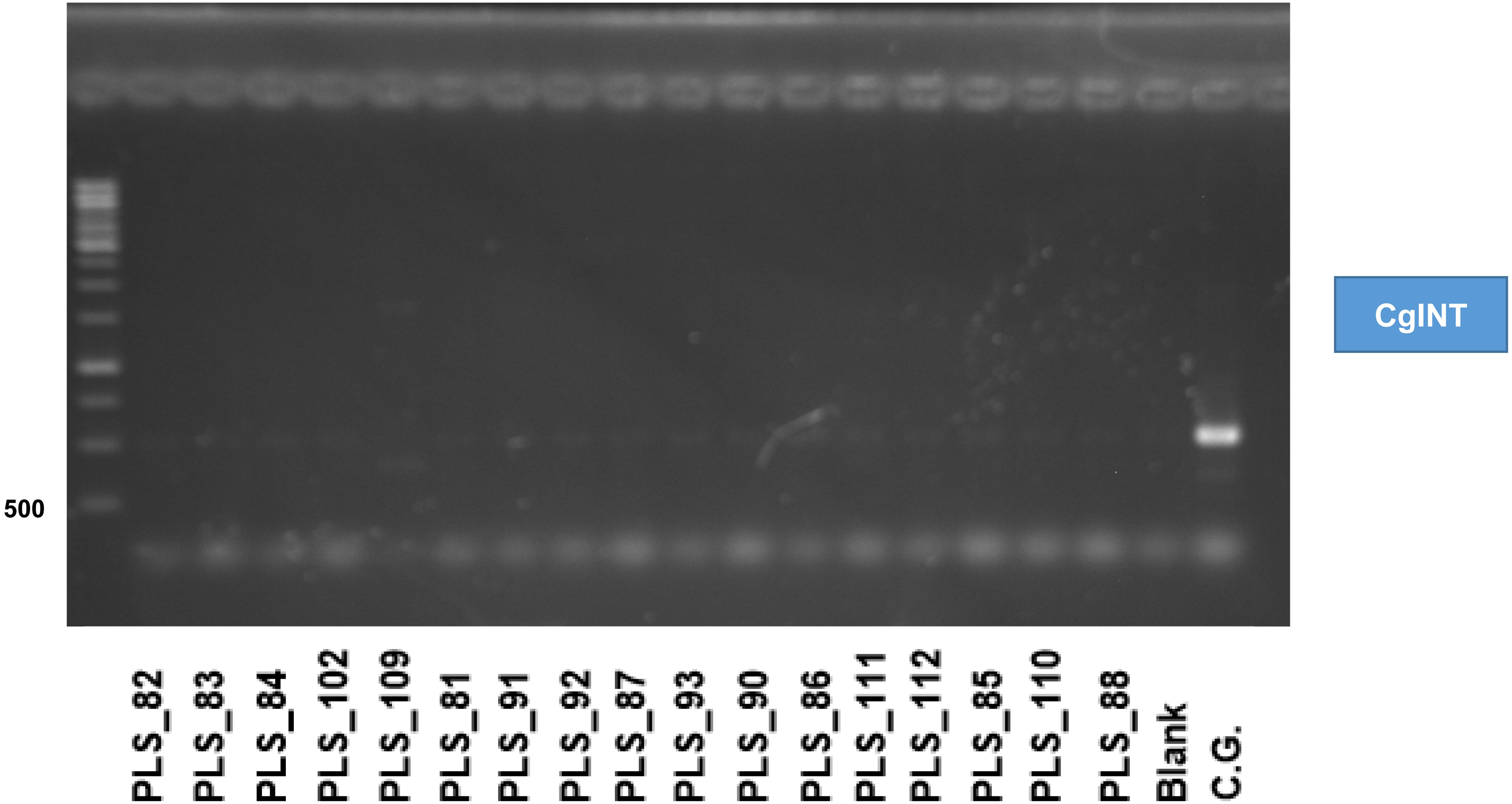

PCR products
1% agarose gel
110V, 30’
150ng template DNA
Reaction volume 50µl
Thermo scientific PCR kit

Fig 2C

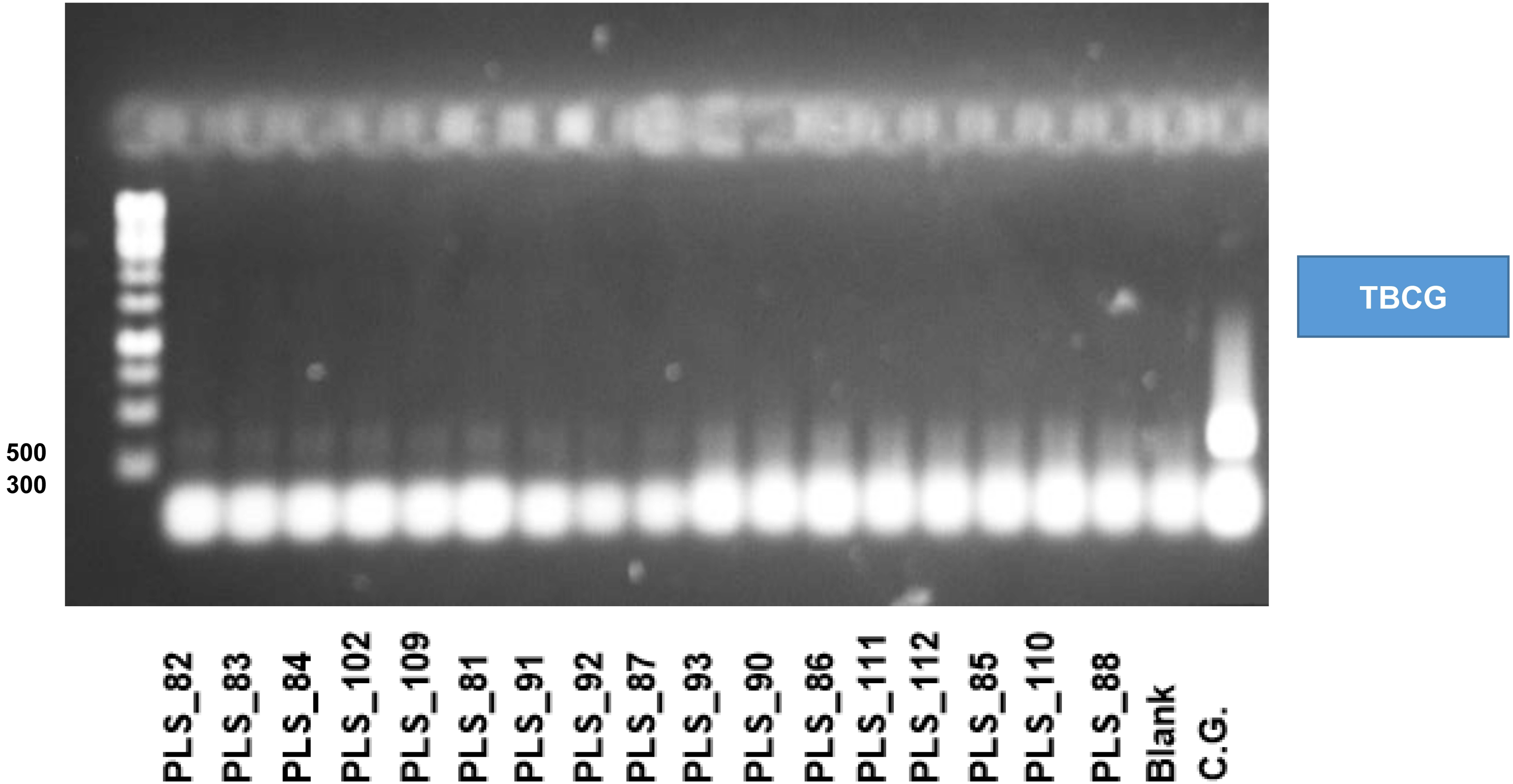

PCR products
1% agarose gel
110V, 30’
150ng template DNA
Reaction volume 50µl
Thermo scientific PCR kit

Fig 2D
